# Quantification of Nanoplastics and Inorganic Nanoparticles via Laser‐Induced Breakdown Detection (LIBD)

**DOI:** 10.1002/smtd.202402060

**Published:** 2025-04-08

**Authors:** Minh N. Nguyen, Pia Lipp, Ines Zucker, Andrea I. Schäfer

**Affiliations:** ^1^ Institute for Advanced Membrane Technology (IAMT) Karlsruhe Institute of Technology (KIT) Hermann‐von‐Helmholtz‐Platz 1 76344 Eggenstein‐Leopoldshafen Germany; ^2^ TZW: DVGW‐Technologiezentrum Wasser Karlsruher Str. 84 76139 Karlsruhe Germany; ^3^ School of Mechanical Engineering Faculty of Engineering Tel Aviv University Tel Aviv 69978 Israel

**Keywords:** engineered nanoparticles, microplastics, online water quality monitoring, particle counting, soft versus hard nanoparticles

## Abstract

Nanoparticles with diverse characteristics are difficult to quantify at low concentrations in the water environment (10^6^–10^9^ particles mL^−1^ for nanoplastics originating from the breakdown of plastic debris) for the evaluation of effective treatment methods. This study examines the sensitivity, or limit of detection (LOD), of laser‐induced breakdown detection (LIBD) for the counting of nanoparticles, including nanoplastics. For polystyrene (PS) standards with sizes of 20−400 nm, LIBD shows relatively low LODs (for example, 2 × 10^6^ particles mL^−1^ for 100 nm particles) compared with turbidity monitoring, UV–vis spectroscopy (both 6 × 10^8^ particles mL^−1^), and nanoparticle tracking analysis (2 × 10^7^ particles mL^−1^). For nanoplastics (PS, polypropylene, and polyethylene terephthalate), the detection limits are 10^4^ − 10^5^ particles mL^−1^, one to two orders of magnitude lower than the PS standards. LIBD can quantify inorganic nanoparticles, such as zeolite, titania, and hematite. The sensitivity increases (i.e., LOD reduces) with increasing particle density, while some particles are prone to artifacts. The low LODs make LIBD a robust technique for counting nanoparticles of various types and sizes, even at the concentrations found in the permeate of membrane‐based water treatment systems. Given the high sensitivity, LIBD has the potential to be applied in membrane integrity monitoring and fundamental studies on membrane mechanisms.

## Graphical abstract

Laser‐induced breakdown detection (LIBD) is a highly‐sensitive technique to quantify nanoparticles, including nanoplastics, in clean water. The limit of detection for particle concentration is as low as 10^4^ − 10^6^ particles mL^−1^ and is influenced by particle properties, notably the particle density. LIBD has the potential to be applied in membrane integrity monitoring and fundamental studies on membrane mechanisms.

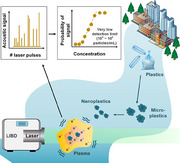

## 1 Introduction

**Figure 1:**
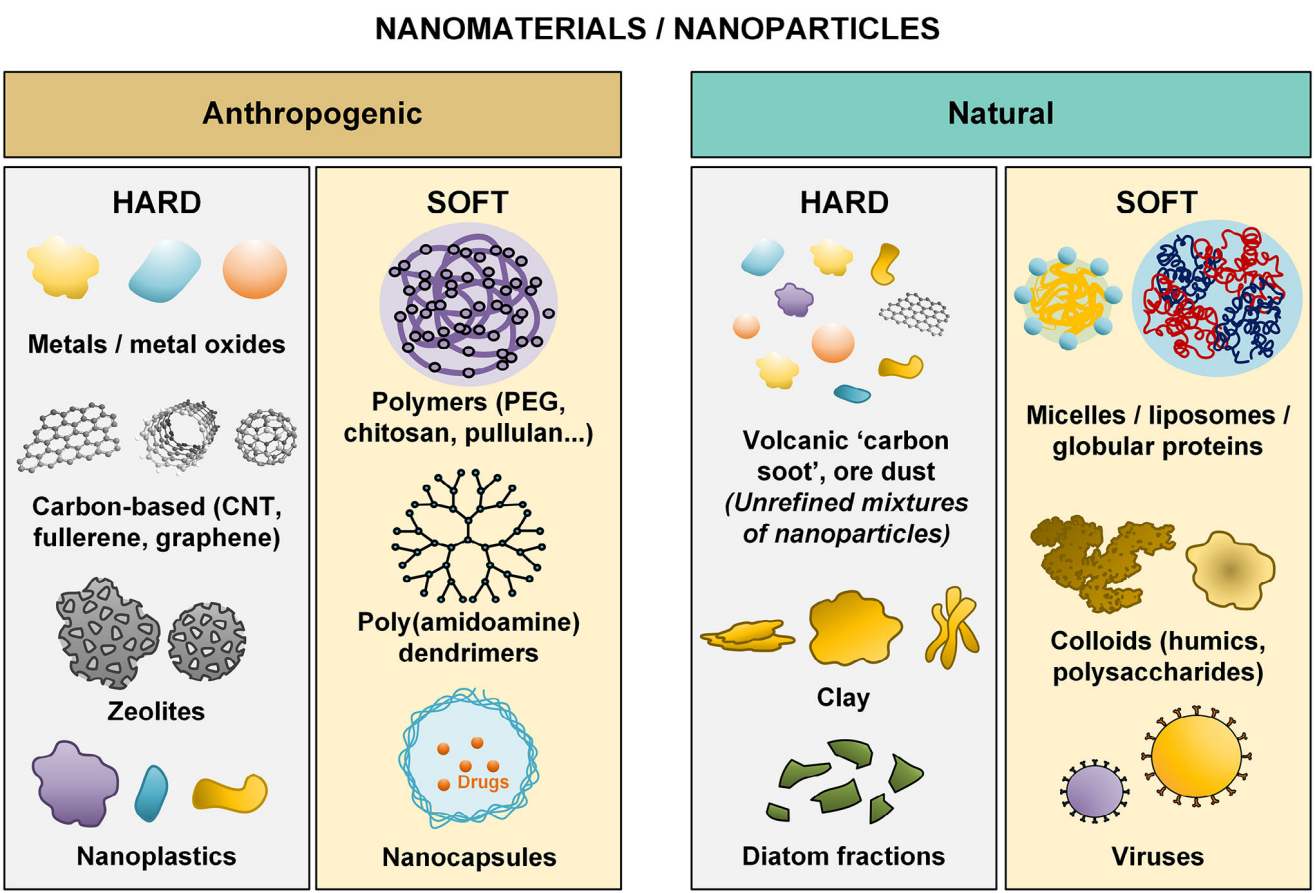
Nanomaterial (NP) types are categorized into soft and hard based on deformability.^[^ ^8^, ^9^, ^10^ ^]^

Nanoparticles (NPs) are particles of any shape with sizes in all dimensions ranging from 1 to 100 nm,^[^ ^1^, ^2^ ^]^ although the prefix “nano‐” is practically accepted for extended sizes of up to 500 nm, or tubes/fibers with two of the dimensions below 100 nm.^[^ ^1^ ^]^ According to this definition, NPs include a wide of materials, such as engineered products designed for diverse applications (water treatment, energy, sensors, cosmetics, electronics and biomedicine),^[^ ^3^ ^]^ degradation products from larger materials (i.e., the case of nanoplastics),^[^ ^4^ ^]^ biological components in the body (micelles, liposomes, and proteins),^[^ ^5^ ^]^ natural colloids,^[^ ^6^ ^]^ and viruses^[^ ^5^, ^7^ ^]^ (see **Figure** **1**). Based on deformability, NPs can be classified into hard and soft NPs.^[^ ^8^, ^9^, ^10^ ^]^ Hard NPs (such as metals, metal oxides including zeolites, and carbon‐based NPs) contain high densities of atoms in their structures and are not deformable. For polymer‐based NPs, including nanoplastics, deformability is linked with the degree of crosslinking.^[^ ^11^, ^12^ ^]^ Soft polymeric NPs (such as nanospheres, nanocapsules, and dendrimers used for drug transport, proteins, and natural colloids) are characterized by a relatively low degree of crosslinking and high void volumes, i.e., a small mass of the polymer molecule(s) occupying a relatively large volume in the water matrix. The physical characteristics of these soft NPs, namely the hydrodynamic size and conformation, depend on the surrounding water environment, such as temperature, ionic strength, and pH.^[^ ^13^, ^14^, ^15^ ^]^ Both hard and soft NPs can agglomerate in water, leading to variations in size and polydispersity over time.^[^ ^15^, ^16^, ^17^ ^]^

Nanoplastics produced from various degradation processes of plastics in the environment^[^ ^18^, ^19^ ^]^ are an important removal target of drinking water treatment. At nanoscale sizes (<1000 nm), all nanoplastics suspend in water and exhibit colloidal behavior.^[^ ^18^ ^]^ Originated from plastics and microplastics with high densities (between 0.8 and 1.4 g mL^−1^, close to the bulk densities of the respective plastics),^[^ ^20^, ^21^, ^22^ ^]^ nanoplastics are considered hard NPs. Nanoplastics can enter the aquatic food chain and adhere to the surfaces of cells and tissues of aquatic organisms.^[^ ^23^, ^24^ ^]^ As a result, nanoplastics can accumulate in the intestines, livers, and even the circular systems and brains of marine organisms^[^ ^25^ ^]^ and the human body. While the majority of nano‐ and microplastics are likely excreted by the intestinal system,^[^ ^26^ ^]^ disruption of metabolism and reproduction in aquatic organisms has been reported at environmentally relevant concentrations (0.3−20 µg L^−1^).^[^ ^27^, ^28^ ^]^ The mass concentration of nanoplastics in surface water ranges between 0.3 and 500 µg L^−1^.^[^ ^29^, ^30^, ^31^ ^]^ Assuming an average sphere diameter of 100 nm and nanoplastic density of about 1 g mL^−1^, the above mass concentration range translates to a range of particle number of 10^6^–10^9^ particles mL^−1^. Because of the varied shapes, sizes, and types of nanoplastics, as well as the complex composition of the water matrices, it is challenging to detect and quantify nanoplastics in the water environment.^[^ ^32^ ^]^ For environmental research, nanoplastics can be fabricated in the laboratory via growth from pre‐cursors,^[^ ^33^ ^]^ or crushing and weathering of plastics and microplastics.^[^ ^34^, ^35^ ^]^ The former technique produces spherical nanoplastics that do not reassemble real nanoplastics, while the nanoplastics produced using the later technique are of higher environmental relevance.

To determine the size and concentration (for example, in particles per milliliter) of NPs, including nanoplastics, several techniques have been deployed as summarized in Table S1 (Supporting Information). Dynamic light scattering (DLS) and single particle counting are commonly used techniques based on the principle of light scattering. Reliable single particle counting is limited to ≥100 nm NP sizes,^[^ ^36^, ^37^ ^]^ while DLS requires high concentrations of NPs (corresponding to mass concentrations in the order of 10 mg L^−1^) for detecting the light scattering signals.^[^ ^38^ ^]^ Multi‐angle light scattering^[^ ^39^ ^]^ and nanoparticle tracking analysis (NTA)^[^ ^40^, ^41^ ^]^ are state‐of‐the‐art techniques that offer relatively low particle concentration detection limits (LODs) of 10^7^ − 10^8^ particles mL^−1^.

Scanning/transmission electron microscopy (SEM/TEM) and atomic force microscopy are sensitive for NP size determination down to 0.2−1 nm, but they require the NPs to be extracted from environmental matrices.^[^ ^42^, ^43^ ^]^ Microscopy can be combined with other spectroscopic analyses, such as Fourier‐transform infrared (FTIR) and Raman spectroscopy. The resulting micro (μ)‐FTIR and μ‐Raman can characterize both the size and surface chemistry of particles and NPs, including micro‐ and nanoplastics.^[^ ^44^ ^]^ μ‐FTIR is only suitable to characterize microplastics with sizes >10–20 µm^[^ ^44^ ^]^ while μ‐Raman can characterize smaller nanoplastics that are 50−400 nm in size.^[^ ^45^, ^46^ ^]^

Mass spectrometry (MS) techniques, such as single‐particle ion‐coupled plasma (spICP‐MS) and pyrolysis − gas chromatography (Pyr‐GC‐MS), determine the bulk composition of NPs and, from that, the particle characteristics such as size and concentration. spICP‐MS is useful to quantify metallic and metal oxide NPs^[^ ^47^ ^]^ with particle concentrations as low as 10^6^ particles mL^−1^. spICP‐MS has also been deployed to detect metal‐doped micro‐ and nanoplastics with sizes as low as 135 nm and particle count LODs also in the order of 10^6^ particles mL^−1^.^[^ ^48^ ^]^ Without the metal dopes, microplastics with sizes as low as 1.2 − 5 µm and concentrations as low as 10^3^ particles mL^−1^ can be detected with spICP‐MS via ^13^C isotope monitoring.^[^ ^49^, ^50^ ^]^ Py‐GC‐MS is the state‐of‐the‐art technique to quantify nanoplastics.^[^ ^31^, ^51^ ^]^ With reported mass concentration LODs of 0.04−0.44 µg L^−1^,^[^ ^51^ ^]^ Py‐GC‐MS is suitable for detecting nanoplastics at relevant concentrations in surface water (0.3−500 µg L^−1^).

Among all techniques listed in Table S1 (Supporting Information), LIBD allows in‐line monitoring^[^ ^52^, ^53^ ^]^ and offers the lowest LODs, in the order of 10^5^ − 10^6^ particles mL^−1^ for polystyrene standards.^[^ ^54^, ^55^ ^]^ These are in the lower end of nanoplastic concentrations in surface water (10^6^–10^9^ particles mL^−1^). Therefore, LIBD can be used for quantifying nanoplastics or nanoplastic surrogates at realistic concentrations, with potential applications in water quality assessment and pollutant monitoring.

**Figure 2:**
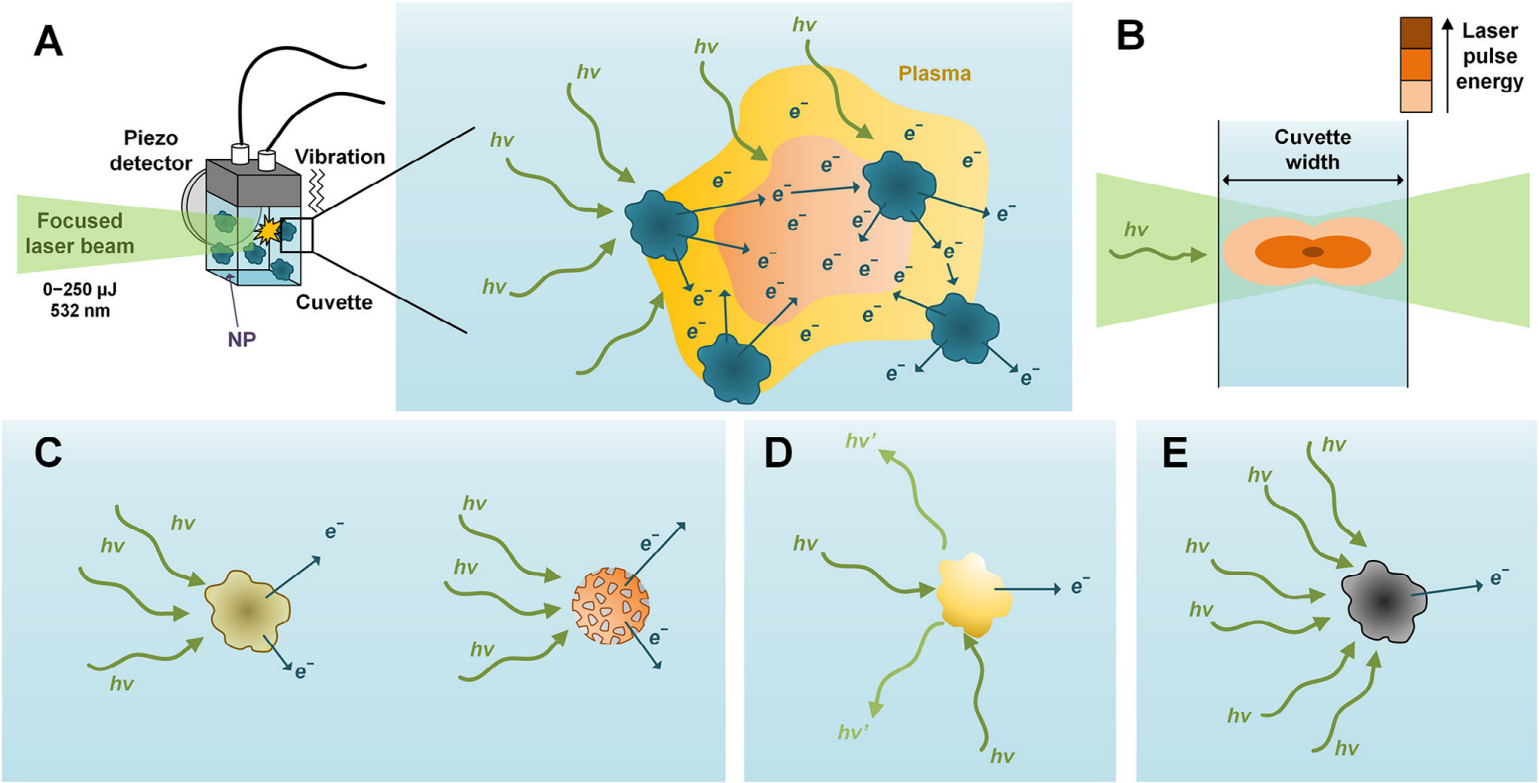
A) Simplistic view of acoustic LIBD operation, where the laser beam ionizes the NPs forming a plasma (i.e., dense area of excited electrons) and resulting in a detectable shockwave signal. B) Schematic of the focal volume (i.e., the volume irradiated by the focused laser beam) and distribution of laser pulse energy. C, D) Major characteristics of NPs that affect LIBD sensitivity: softness/particle density/particle porosity (B), reflectiveness (C), and ionization energy (D).

In LIBD, the samples are irradiated with a focused laser beam (e.g., green laser with wavelength 515−532 nm and energy as high as several millijoules), causing NPs in the samples to ionize. The excited electrons then gain energy from the laser irradiation − the process is called *inverse Bremsstrahlung* – to accelerate, and then collide with other NPs, releasing more electrons by ionization.^[^ ^56^ ^]^ Once a lot of electrons are excited in a dense volume within nanoseconds (10^18^ electrons mL^−1^), a plasma is formed^[^ ^57^, ^58^ ^]^ and detected either with a charged‐coupled device camera (optical detection), or a piezo crystal (acoustic detection) as a plasma shockwave (**Figure** **2**A) is generated.^[^ ^54^ ^]^ The optical detection reveals the positions of the plasma plumes (i.e., the NPs); based on the plume characteristics, the size and concentration of NPs can be determined.^[^ ^59^ ^]^ On the other hand, the acoustic detection records only the “noise” caused by the shockwave.^[^ ^60^ ^]^ With both detection methods, the breakdown probability (BDP) is reported, which is the number of positive signals (plumes or shockwaves) per a fixed number of laser pulses. At low laser pulse energies (i.e., low laser power densities), the excited electrons (if any) in the focal volume (illustrated in Figure 2B) are below the required quantity to trigger plasma formation,^[^ ^57^, ^61^ ^]^ i.e., BDP = 0. A laser pulse energy threshold can be identified, above which the plasma begins to form and the BDP increases to a value above zero. As the laser energy increases further, the probability of a plasma being detected increases and hence the higher BDP (until the BDP reaches unity).^[^ ^57^ ^]^ With acoustic detection, BDP = 1 means that all the laser pulses result in positive acoustic signals.

Due to the required *en masse* ionization, the LIBD sensitivity depends on the size and concentration of NPs, as well as characteristics such as the ionization energy, size, optical property (light scattering property and transparency), and aggregation state (Figure 2C−E). The particle density (degree of branching and crosslinking of polymeric materials^[^ ^62^ ^]^ or packing of atoms in the crystal structures of inorganic NPs^[^ ^63^ ^]^) is an important characteristic. The higher the areal density of atoms in the particle, the lower the laser energy required to trigger plasma formation (with a density of electrons of 10^18^ mL^−1^). As the ionization becomes less effective when the density of solid masses is low, the sensitivity of LIBD toward soft NPs will be lower than hard NPs.

An important characteristic of NPs is the ionization energy (IE), which is the energy required to remove an electron from the ground state of an atom or a molecule in the gas phase.^[^ ^64^ ^]^ In atoms, the IE depends on the atomic structure; however, in molecules, the IE is an indicator of bond strength.^[^ ^65^, ^66^ ^]^ As the photon energy (e.g., 2.3 eV when the photons are emitted with a 515−532 nm green laser) is lower than the IEs of most solids (>6 eV),^[^ ^67^ ^]^ the simultaneous energy transfer from multiple photons are required to form a single ionization event − the phenomenon is called multi‐photon ionization (MPI).^[^ ^57^, ^68^ ^]^ The stronger the bonds in NPs, the higher the IEs; hence fewer photons have sufficient energy for ionization. As a result, the plasma formation probability and LIBD sensitivity are lower. If MPI limits breakdown generation, a rise in BDP following the power law (≈4 orders for polystyrene) with laser pulse energy can be observed.^[^ ^69^, ^70^ ^]^ However, at a relatively high laser energy, the thermal emission of electrons (i.e., generation of electrons from the heating of NPs by laser) becomes significant.^[^ ^71^, ^72^ ^]^ In consequence, plasma formation is no longer limited by MPI, but by the probability of finding the NPs in the focal volume (power order of 1). A “break” in the BDP pattern at a laser energy of 600 µJ has been found for polystyrene hard NPs with a particle size of 100 nm.^[^ ^70^ ^]^

Particle quantification with LIBD has only yielded consistent results when the NPs are suspended in pure water or simple water matrices. Water chemistry (pH, ionic strength, and interferants, e.g., natural colloids and surfactants) influences the size, aggregation state, and colloidal stability of NPs, and hence the BDP can be varied.^[^ ^55^, ^73^, ^74^ ^]^ Additionally, when NPs are present in various sizes (i.e., in a multimodal suspension), the breakdown signals of larger NPs will mask the signals of smaller NPs and make NP quantification challenging.^[^ ^75^, ^76^ ^]^ Hence, LIBD needs to be coupled with a size separation technique (e.g., flow field‐flow fractionation) to acquire concentration information of one particle type at a time.^[^ ^77^, ^78^ ^]^ It is important to note that LIBD calibration has primarily been carried out with PS standards; for unknown NPs and colloids, the BDP is usually correlated to the calibration with PS standards to estimate the NP/colloid concentration.^[^ ^55^, ^60^ ^]^ This correlation is problematic because the BDP results are specific for the NP type, of which characteristics such as particle density, shape, composition, and optical properties may differ significantly from PS standards.

Because LIBD has not been applied to quantify environmentally relevant nanoplastics, the sensitivity to three types of nanoplastics will be systematically investigated and compared to the sensitivity to i) spherical PS standards NPs, ii) soft polymeric “NPs” (polystyrene sulfonate), and iii) inorganic NPs with varied particle densities. The main research questions are: How does the limit of detection (LOD) attained with LIBD compare with other particle counting techniques for PS standards? How does the LOD for inorganic and organic NPs vary with particle density? What are the LODs for nanoplastics in comparison to those for PS standards?

## 2 Results and Discussion

First, the sensitivity of LIBD was evaluated by developing the calibration method using PS standards (particle sizes 20−400 nm and particle density 1.05 g mL^−1^), with varied mass concentrations ranging from 30 ng L^−1^ to 30 mg L^−1^. The LODs attained were then compared to those of other particle quantification techniques, such as NTA, UV–vis spectroscopy, and turbidity tests. Subsequently, the capability of LIBD at detecting (low‐density) polymers was assessed with PS sulfonate polymer standards (hydrodynamic sizes 41 and 142 nm, and solid density 0.006 g mL^−1^). The sensitivity of LIBD was examined for hard inorganic NPs with an order of magnitude variation of particle density (0.5−5.2 g mL^−1^), and finally with several types of environmentally relevant nanoplastics (particle sizes ≈100 nm, particle densities 0.9−1.4 g mL^−1^).

### 2.1 Polystyrene Standard Detection at Varied Laser Pulse Energies

**Figure 3:**
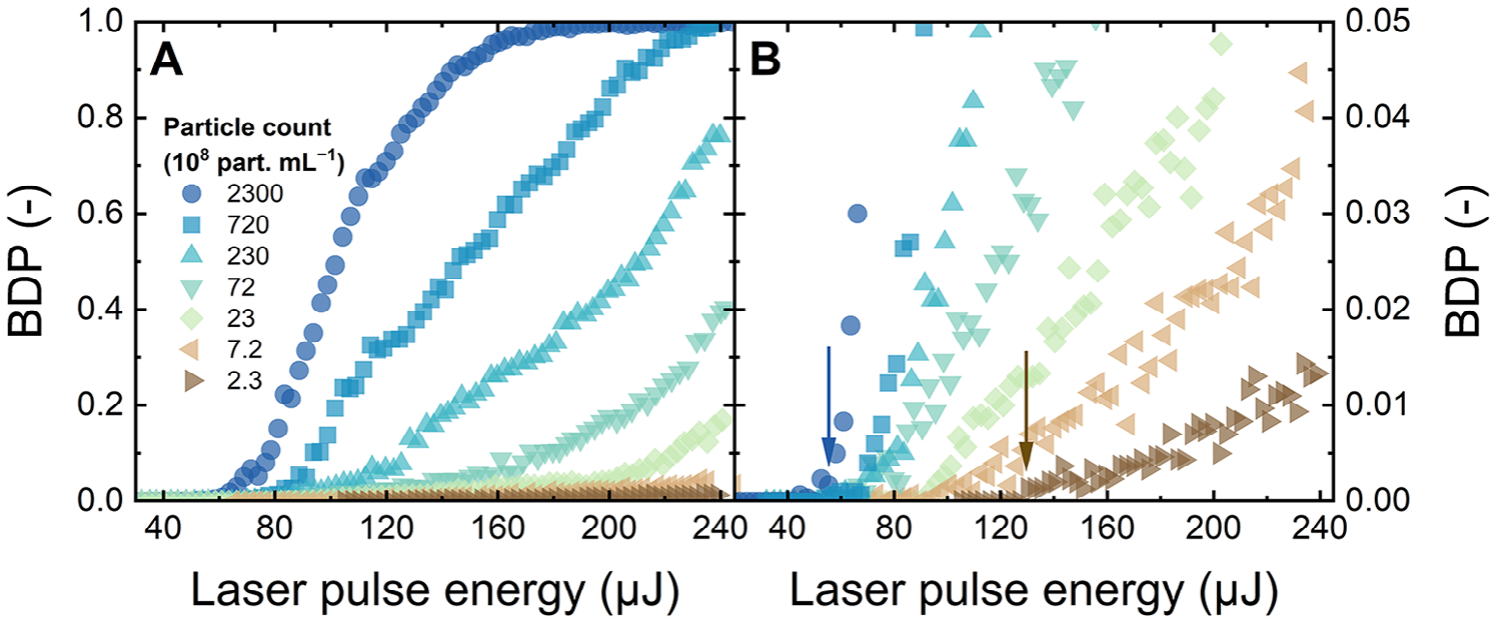
BDP versus laser pulse energy of PS 20 nm at different concentrations (2.3 × 10^8^ − 2.3 × 10^11^ particles mL^−1^), corresponding to the mass concentration range of 0.001−1 mg L^−1^. (B) is the zoom‐in of (A). The blue and brown arrows in (B) indicate the energy thresholds at the highest and lowest concentrations, respectively. Flow rate 1 mL min^−1^, 1 mM NaHCO_3_, 10 mM NaCl, 23 ± 2 °C, pH 8.2 ± 0.1.

To determine how the laser energy (or laser power density) impacts the probability of acoustic signals being detected, the BDP for PS 20 nm (given as an example) was plotted against the laser pulse energy varying from 30 to 240 µJ as shown in **Figure** **3**. For clarity, the zero BDP data at laser pulse energies below 30 µJ are omitted from the figure.

From Figure 3A, at the higher concentrations of 7.2 × 10^9^ − 2.3 × 10^11^ particles mL^−1^, the BDP – determined as the ratio of the number of signals that surpasses a pre‐defined acoustic level to the number of laser pulses, see Figures S1 and S2 (Supporting Information) − increased to a value above zero (for example, 0.001) at around 57 ± 5 µJ. This energy threshold is where the first events of plasma formation were detected. When the laser pulse energy was above this threshold (i.e., in the range of 60–240 µJ), the BDP increased with increasing laser pulse energy until the BDP reached unity, forming an S‐curve shape. The energy threshold (indicated by the arrows in Figure 3B) was uniform at 58−62 µJ at the higher concentrations (2.3 × 10^11^ − 2.3 × 10^10^ particles mL^−1^, but then shifted toward higher energy values, from 58 to 130 µJ, as the concentration decreased from 2.3 × 10^11^ to 2.3 × 10^8^ particles mL^−1^. This shift is because the probability of the NPs entering the focal volume of LIBD reduced with decreasing concentration, hence it was less likely to trigger a single event of plasma formation. From Figure 3A,B, at the same laser energy above the energy threshold(s), the higher the particle concentration, the higher the BDP. It is shown in Figure S15 (Supporting Information) that similar increases in BDP with increasing laser pulse energy and particle concentration were observed for other particle sizes (50–400 nm). Figures S15 and S16 (Supporting Information) reveal that, as the standard particle size increased from 20 to 400 nm, there was a slight decreasing trend in laser pulse energy, although the differences between the particle sizes of 150, 200, 300, and 400 nm were not clear. The larger the particles, the higher the PS mass in the focal volume that is irradiated by the laser to trigger plasma formation events, and the lower the energy threshold.^[^ ^57^ ^]^ The same phenomenon had been observed in previous studies with the LIBD.^[^ ^54^, ^79^ ^]^

Since the BDP generally increases with increasing laser energy, the laser pulse energy will be fixed at the highest level (240 µJ) to maximize NP detection sensitivity at low concentrations. In theory, the sensitivity of LIBD can be enhanced by further increasing the laser energy beyond 240 µJ – until the breakdown signals of water are registered.^[^ ^54^ ^]^ Next, the LODs attainable with LIBD will be determined by varying the particle concentrations.

### 2.2 Detection Limits of LIBD for Polystyrene Standards

**Figure 4:**
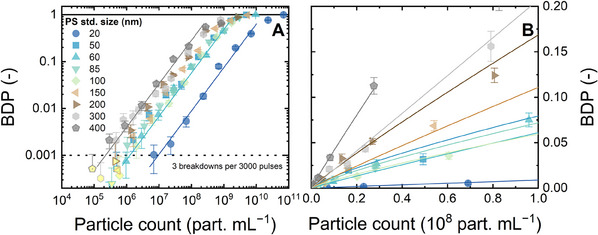
A) BDP (at a laser pulse energy of 240 µJ) versus particle concentration for various PS standard sizes. All the LODs were found just above a threshold indicated with a vertical dotted line (BDP = 0.001). Error bars are standard deviations from 40 measurements. Data points below the LOD are filled with yellow color. B) Representation of A in linear scale up to a concentration of 10^8^ particles mL^−1^. Flow rate 1 mL min^−1^, 1 mM NaHCO_3_, 10 mM NaCl, 23 ± 2 °C, pH 8.2 ± 0.1.

To determine the detection ranges and LODs attained with the LIBD, the relationship between the BDP and particle count of PS 20–400 nm standards was determined as shown in **Figure** **4**. From multiple repeats per concentration, a normal distribution of BDP could be determined (Figures S3 and S4, Supporting Information), and the error bars shown in Figure 4 are the standard deviations.

From Figure 4A, a linear regression can be established to link BDP (no units) with particle count (in particles mL^−1^) in the double logarithmic scale, with BDP varying between 0.002 and 0.4−0.6. Above this range, i.e., when the BDP was closer to unity, the BDP flattened out with increasing particle concentration. This is because the quantity of released electrons, which should scale with the quantity of solid mass in the laser focal volume, was abundant and no longer limited plasma formation; hence, a shockwave signal caused by the plasma was likely recorded with each laser pulse. The slope of the regression in the double logarithmic scale was relatively uniform for all PS standards and close to unity (0.9 ± 0.1), see Table S2 (Supporting Information). This translates into a relationship that is close to linear in the normal scale (Figure 4B).

With other analytical techniques, the detection signals typically deviate from the regression at concentrations close to or below the detection limit (LOD), making it impossible to differentiate these concentrations from the calibration. This was not observed with LIBD, because at concentrations below the LOD, the BDP was zero, and no plasma was formed or detected. For a data point at a very low concentration (below LOD), in 40 repeat measurements, a large number of measurements (>10) returned a BDP equal to 0. The remaining measurements returned very low BDPs but these were greater than 0, typically corresponding to 1–2 breakdown events per 3000 laser pulses (Figure S5, Supporting Information). The LOD was then defined as the particle count below which the standard deviation of BDP is larger than the average BDP. With this definition, the LODs were 7 × 10^6^ particles mL^−1^ (0.03 µg L^−1^) for PS 20 nm, (1.5−3) × 10^6^ particles mL^−1^ (0.1−5 µg L^−1^) for PS 50−200 nm, and (3–5) × 10^5^ particles mL^−1^ (7−10 µg L^−1^) for PS 300 and 400 nm. These LODs are in the same order as those reported with the acoustic LIBD variant, namely, 3 × 10^6^ for PS 20 nm and 7 · 10^4^ for PS 500 nm standards.^[^ ^55^ ^]^ In Figure 4A, all the LODs were found just above a threshold indicated with a vertical dotted line (BDP = 0.001 or three breakdown signals detected per 3000 incident laser pulses). With the upper limit for NP detection of 10^8^–10^10^ particles mL^−1^, LIBD can detect NPs at concentrations spanning 3–4 orders of magnitude in high purity background electrolytes. This is particularly useful in membrane integrity monitoring, where a 3–4 log removal of probe species is expected for highly integrated ultrafiltration membranes.^[^ ^80^, ^81^ ^]^

### 2.3 Comparison of Sensitivity Toward Polystyrene Standards of Several Counting Techniques

**Figure 5:**
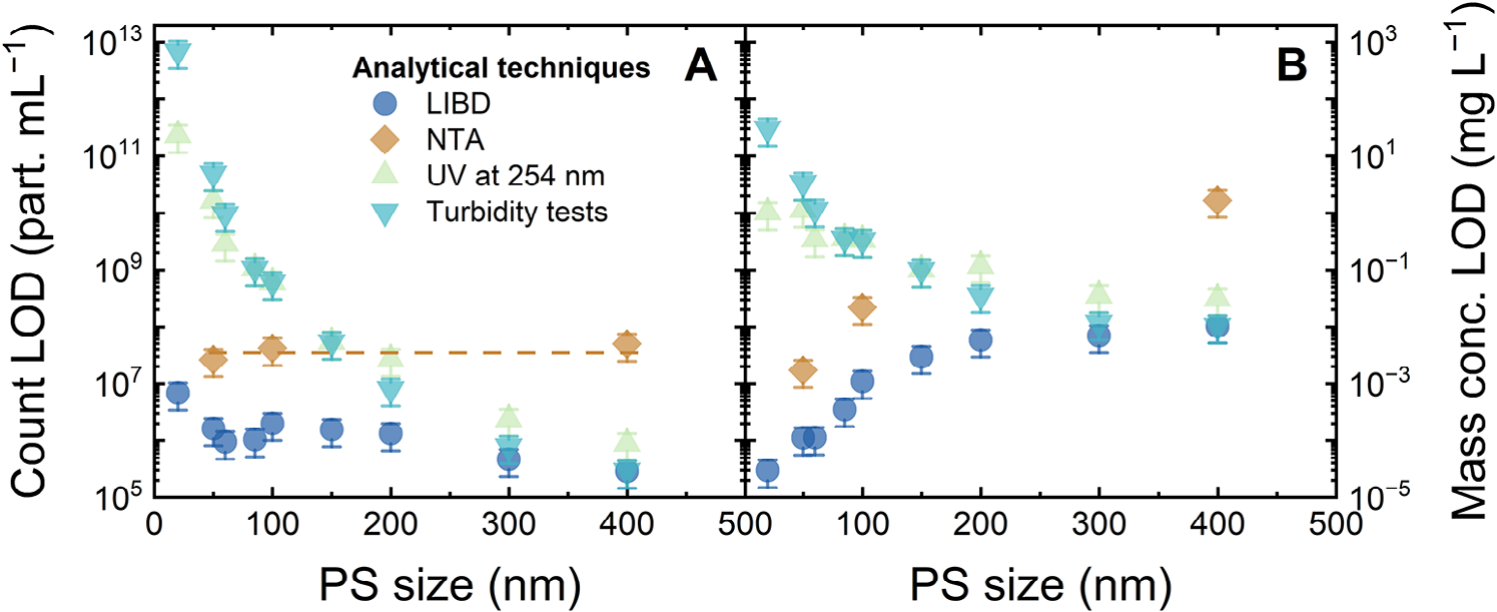
Limit of detection (LOD) of LIBD for both particle count (A) and mass concentration (B) in comparison with other techniques: UV–vis spectroscopy at 254 nm, turbidity measurement, and nanoparticle tracking analysis (NTA). 1 mM NaHCO_3_, 10 mM NaCl, 23 ± 2 °C, pH 8.2 ± 0.1.

To emphasize the high sensitivity of LIBD, the LOD of LIBD was compared to the LODs of other particle counting methods, namely UV–vis spectroscopy (because PS absorbs light at UV wavelengths, see Figure S7, Supporting Information), turbidity measurements, and NTA, as shown in **Figure** **5**. The logarithmic relationship between UV absorbance at 254 nm/turbidity and PS particle count is given in Figures S8 and S9 (Supporting Information). The particle counts measured with NTA for the 50, 100, and 400 nm standards are given in Figure S10 (Supporting Information) with a relatively narrow range of 10^7^–10^9^ particles mL^−1^.

Generally, the LODs of UV–vis and the related turbidity techniques (both in particles mL^−1^, see Figure 5A and in mg L^−1^, see Figure 5B) decreased by 5–7 orders of magnitude with increasing particle size from 20 to 400 nm, which means UV based techniques were less sensitive for the detection of small NPs. With turbidity measurements, the mass concentration of 50 and 100 nm NPs were 3000 and 300 µg L^−1^. Because nanoplastics exist in the water environment at a lower concentration (0.3–500 µg L^−1^), turbidity is not sensitive enough for the analysis of NPs at concentrations relevant to nanoplastics. The mass concentration LODs with UV–vis spectroscopy were 1 and 0.3 mg L^−1^ for the NP sizes of 50 and 100 nm, respectively. These high LODs mean that UV‐based techniques are not practical.

The particle number LODs of NTA shown in Figure 5A were relatively uniform at (3−5) × 10^7^ particles mL^−1^ for different NP sizes (50, 100, and 400 nm). These LODs of NTA depend on the level of background noise (10^7^ particles mL^−1^). Below this level, the NPs are not present within the field of view of NTA in quantity that allows statistical evaluation of motion tracking.^[^ ^40^ ^]^ For small NPs (50 and 100 nm), the respective mass concentration LODs of NTA were 2 and 20 µg L^−1^ (Figure 5B). This means NTA is much more sensitive than UV‐based techniques and can quantify NPs at concentrations relevant to nanoplastics in the water environment.

LIBD was even more sensitive than the state‐of‐the‐art NTA. For 50 and 100 nm NPs, LIBD could afford LODs in the order of 2 × 10^6^ particles mL^−1^ (Figure 5A), which is 1 order of magnitude lower than the LODs of NTA, at (3–4) × 10^7^ particles mL^−1^. The corresponding mass concentration LODs of LIBD were 0.1 and 1 µg L^−1^ (Figure 5B). For larger NPs, LIBD became less sensitive. Although the particle number LOD decreased, the mass concentration LODs increased with increasing particle size and approached the LODs of UV–vis and turbidity techniques for 300 and 400 nm NPs (10 − 30 µg L^−1^). It is noted that LIBD is capable of quantifying NPs larger than 400 nm (at least to the order of 1 µm), although the mass concentration LOD increases (i.e., the sensitivity reduces) with increasing particle size.^[^ ^60^, ^79^ ^]^

### 2.4 Analysis of Polystyrene Sulfonate Polymer Standards with Ultra‐Low Density

**Figure 6:**
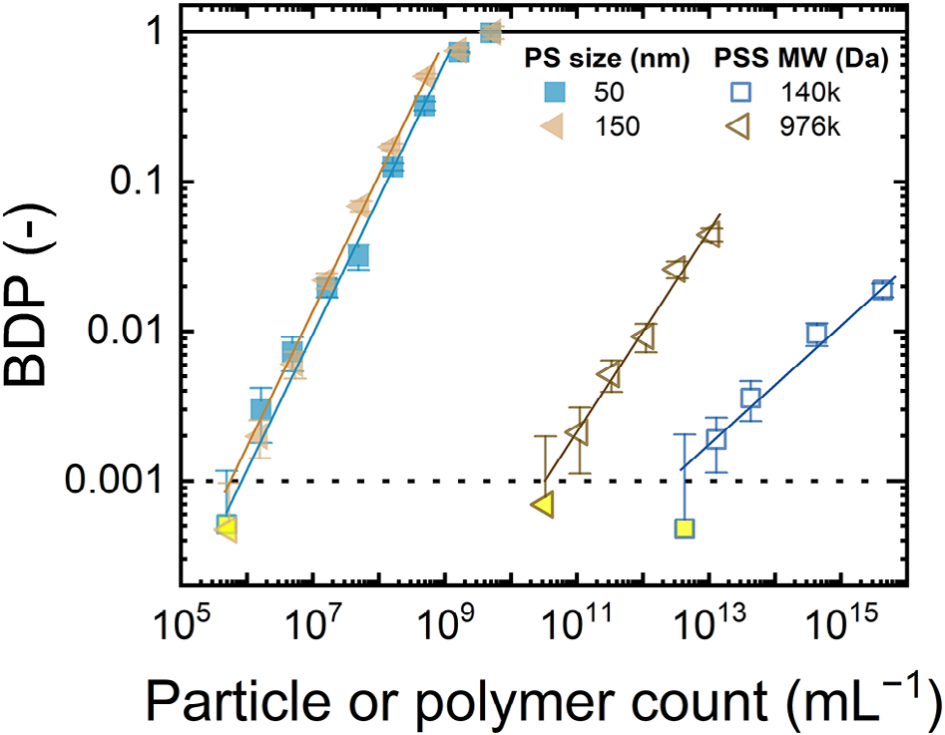
BDP at a laser pulse energy 240 µJ versus particle/polymer count for PS 50 and 150 nm, and polymer number of polystyrene sulfonate (PSS) 140 kDa (equivalent to PS 50 nm) and 976 kDa (equivalent to PS 150 nm). Error bars are standard deviations from 40 measurements. Data points below the LOD are filled with yellow color. Flow rate 1 mL min^−1^, 1 mM NaHCO_3_, 10 mM NaCl, 23 ± 2 °C, pH 8.2 ± 0.1.

LIBD may not work for polymers and colloids, which are categorized under soft “NPs” with very low “density”. To examine the sensitivity of LIBD for low‐density (0.006 g mL^−1^) polymeric NPs, the calibration of LIBD with PSS standards 140 and 976 kDa (hydrodynamic diameters of 41 and 142 nm, respectively) was examined at the highest laser pulse energy (240 µJ) as shown in **Figure** **6**.

A double logarithmic relationship between the BDP and polymer count was observed for soft PSS 140 and 976 kDa polymers (hydrodynamic diameters of 41 and 142 nm, respectively). The particle count LOD is 1.4 × 10^13^ polymers mL^−1^ (mass concentration 3 mg L^−1^) for PSS 140 kDa (41 nm), and 1.1 × 10^11^ particles mL^−1^ (mass concentration 10 mg L^−1^) for PSS 976 kDa (142 nm), much higher than that of the hard PS analogs (below 10^6^ particles mL^−1^ for both PS 50 and 150 nm standards, respectively). This is because a high polymer mass of PSS was required to trigger a plasma (resulting from a high density of electrons), to generate shockwaves and detectable acoustic signals. In summary, LIBD does not detect polymer molecules (i.e., polymeric NPs characterizable with very low densities) and may perform poorly for soft colloids, such as humic acids,^[^ ^82^ ^]^ which is a limitation of this analytical technique. In the next section, LIBD analysis will be examined for hard inorganic NPs (zeolite, titania, and hematite), with particle densities varying between 0.5 to 5.2 g mL^−1^, within an order of magnitude.

### 2.5 LIBD Analyses of Inorganic Nanoparticles with Varied Particle Densities

**Figure 7:**
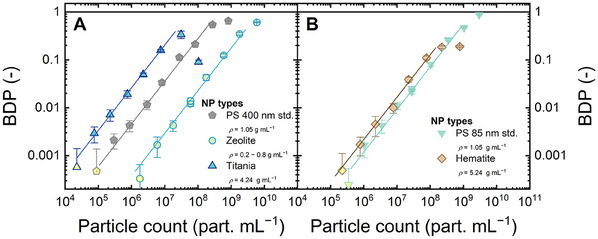
BDP versus particle count of titania and zeolite (A) as well as hematite (B). The calibrations for PS 400 and 85 nm standards are given for comparison. Error bars are standard deviations from 40 measurements. Data points below the LOD are filled with yellow color. Flow rate 1 mL min^−1^, 1 mM NaHCO_3_, 10 mM NaCl, 23 ± 2 °C, pH 8.2 ± 0.1.

To examine the link between LIBD sensitivity and particle density, calibration was carried out with varied inorganic NPs, namely, porous zeolites (0.2−0.8 g mL^−1^, although the intrinsic density is 2.66–2.71 g mL^−1^), titania (4.24 g mL^−1^), and hematite (5.24 g mL^−1^), as shown in **Figure** **7**. The morphology, size distribution, and polydispersity of the inorganic NPs are shown in Figures S11 and S12 (Supporting Information).

For all inorganic NPs, a regression between the BDP and particle count was found in the double logarithmic scale, similar to the case of PS standards. From Figure 7A, the LOD for porous zeolites (particle density of 0.2−0.8 g mL^−1^) was 6 × 10^6^ particles mL^−1^, which is one order of magnitude higher than PS 400 nm standards (2 × 10^5^ particles mL^−1^). This lower sensitivity for zeolites can be attributed to the lower particle density as well as the higher ionization energy of SiO_2_ (12–13 eV), which is the major component of zeolites, compared to PS (7.8 eV). The higher the IE, the more laser photons simultaneously required to generate one ionization event, and the potentially lower sensitivity.

On the other hand, the sensitivity of LIBD increased by increasing the particle density. From Figure 7A, the LOD for titania (density 4.24 g mL^−1^) is 8 × 10^4^ particles mL^−1^, lower than that of the PS 400 nm standards (2 × 10^5^ particles mL^−1^). The IE of titania is similar to that of PS. Figure 7B shows that the LOD of hematite (density 5.24 g mL^−1^, IE simialr to PS and titania) is 2 × 10^5^ particles mL^−1^, several times higher than the LOD of PS 85 nm standards (1 × 10^6^ particles mL^−1^). The sensitivity toward hematite was probably affected by the significant absorption of laser light (Figure S14, Supporting Information). Absorption by NPs with high extinction coefficients (such as hematite) caused attenuation of the laser pulse when it passed through the NPs in the suspension,^[^ ^83^, ^84^ ^]^ which reduces the power density for other ionization events.

In summary, LIBD allows for high‐sensitivity detection of hard NPs (both organic and inorganic) with particle density in the range of 0.5−5.2 g mL^−1^. Hence, in principle, LIBD is suitable for the detection of nanoplastics, of which density varies between 0.9 and 1.4 g mL^−1^. In the next sections, the LODs for nanoplastics will be determined and compared to those for PS standards.

### 2.6 Sensitivity of LIBD Toward Environmentally Relevant Nanoplastics

**Figure 8:**
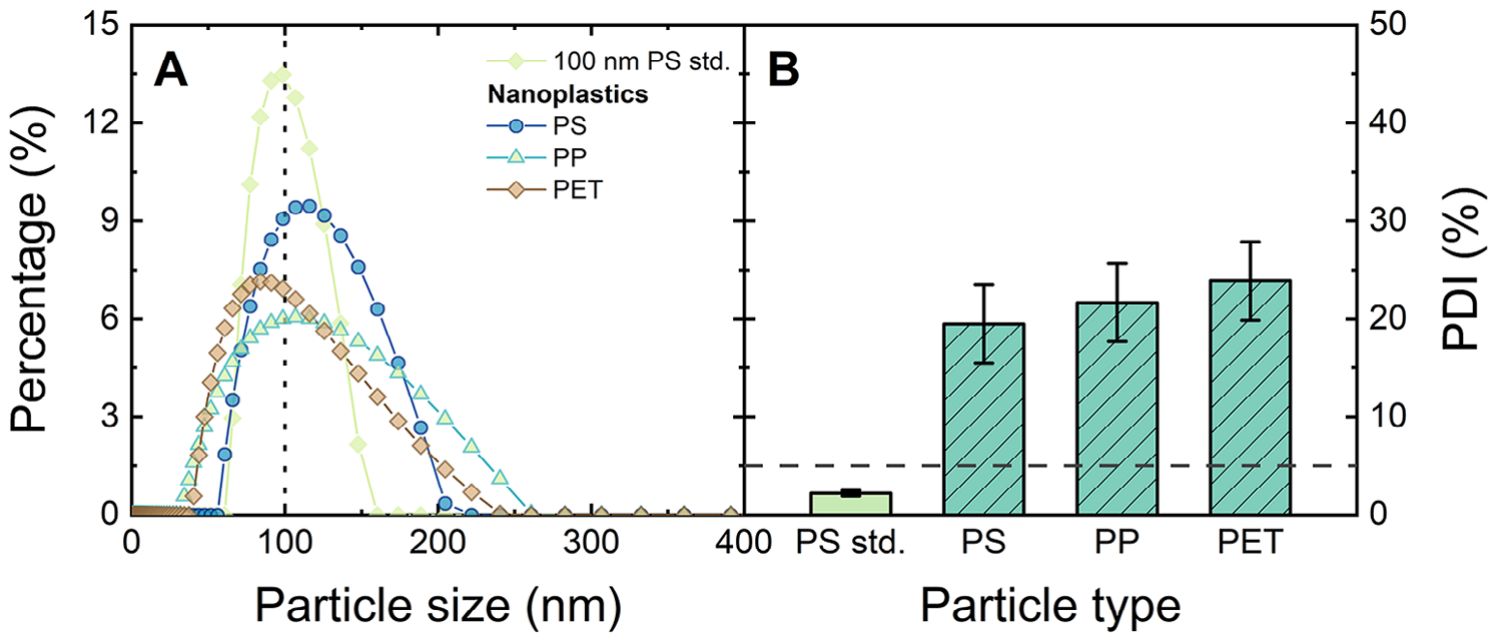
Size distribution (A) and polydispersity indices (PDI) (B) of nanoplastics compared with PS 100 nm standards, measured with DLS. The horizontal dashed line in B indicates the polydispersity threshold. 1 mM NaHCO_3_, 10 mM NaCl, 20 °C.

To determine the average size and size distribution of environmentally relevant nanoplastics, DLS was carried out for nanoplastic samples at 10^9^ − 10^10^ particles mL^−1^ (equivalent to ≈ 0.5–5 mg L^−1^ in mass concentration), and compared to the PS 100 nm standards as shown in **Figure** **8**.

As shown in Figure 8A, the measured size distributions of these nanoplastics were larger than those of the PS 100 nm standards, although the average (modal) sizes of the three nanoplastics were ≈100 nm (PS: 116 nm, PP: 107 nm, PET: 84 nm). The sizes of the nanoplastics varied from 30 to >200 nm. The polydispersity of nanoplastics was also indicated by PDIs between 20 and 24%. A PDI greater than 5% is an indicator of polydispersity.^[^ ^85^ ^]^ Evidently, PS 100 nm standards were monodispersed (PDI < 5%), while the nanoplastics were polydispersed; a potential consequence is that the BDP of nanoplastics may deviate from that of the standard NPs.

**Figure 9:**
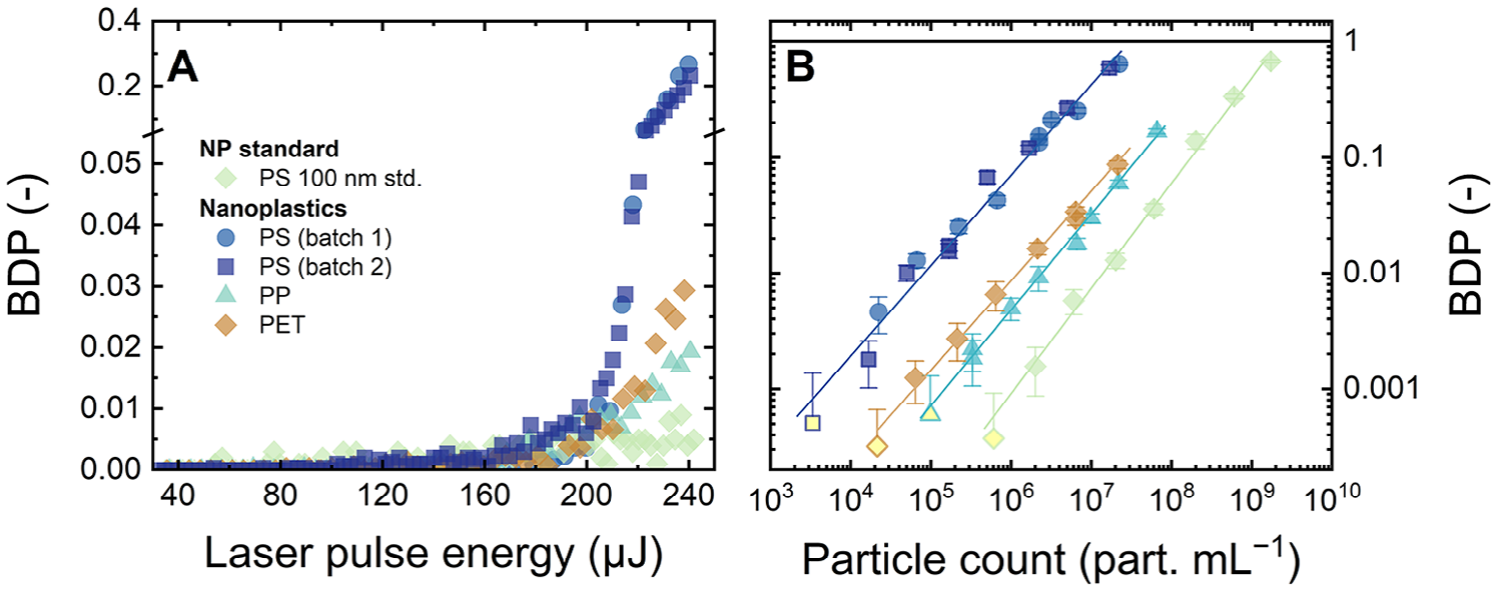
A) BDP versus laser pulse energy at uniform concentrations of (6.0−7.5) × 10^6^ particles mL^−1^. B) Calibration of nanoplastics as BDP versus particle count. Error bars in B are standard deviations from 40 measurements. Data points below the LOD are filled with yellow color. Flow rate 1 mL min^−1^, 1 mM NaHCO_3_, 10 mM NaCl, 23 ± 2 °C, pH 8.2 ± 0.1.

To determine how varying the nanoplastic type affected the breakdown signals, the BDP of nanoplastics was plotted against the laser pulse energy at uniform particle count (6.0−7.5 × 10^6^ particles mL^−1^) as shown in **Figure** **9**A. Higher particle counts were not examined because of the limited amounts of nanoplastics. Figure 9B shows the calibration of nanoplastics at a fixed laser pulse energy of 240 µJ.

As can be seen in Figure 9A, the laser energy thresholds where breakdown signals started were in the range of 140–170 µJ for PS (both the batches), PP and PET nanoplastics. The threshold energy for PS 100 nm standards at the same particle concentration (6.1 × 10^6^ particles mL^−1^) was 57 µJ. At higher concentrations (2.0 × 10^8^–2.0 × 10^9^ particles mL^−1^), the energy threshold was more evident at 42–46 µJ (Figure S15). It appears that the breakdown signals of environmentally relevant nanoplastics were dampened. By increasing the laser pulse energy from 200 to 240 µJ, the BDP of nanoplastics increased sharply. The steepest increase was observed for PS, followed by PET and PP nanoplastics, which indicated higher sensitivity of LIBD toward PS compared to PET and PP at high laser pulse energies.

All the environmentally relevant nanoplastics show lower LODs than PS 100 nm standards (2 × 10^6^ particles mL^−1^), but the LOD of PS was the lowest (1 × 10^4^ particles mL^−1^), followed by PET (6 × 10^4^ particles mL^−1^) and PP (3 × 10^5^ particles mL^−1^), as shown in Figure 9B. The varied LODs could be attributed to many reasons, such as heterogeneity in shape, size, and surface chemistry of nanoplastics, but did not correlate directly with particle density, which varied only slightly between the environmentally relevant nanoplastics (0.9–1.4 g mL^−1^) and the PS standards (1.05 g mL^−1^).

## 3 Conclusion

This work evaluated the sensitivity of LIBD for concentration determination of various NP types, including PS spherical standards, soft polymeric NPs, inorganic NPs with varied particle densities, and nanoplastics of high environmental relevance. The LIBD was operated in‐line, with a uniform flow rate of suspensions of 1 mL min^−1^.

From the calibration with the PS standards (density 1.05 g mL^−1^, sizes 20–400 nm), the LODs of LIBD were relatively low, for example, 2 × 10^6^ particles mL^−1^ for 100 nm particles compared with turbidity tests and UV–vis spectroscopy (6 × 10^8^ particles mL^−1^) and the state‐of‐the‐art NTA (2 × 10^7^ particles mL^−1^). Similar to light scattering techniques, LIBD was not suitable for polymeric NPs such as PSS polymers due to the very low density (0.006 g mL^−1^, i.e., a very large volume of voids surrounding the polymers). Within a higher range of particle density (0.5–5.2 g mL^−1^) of hard inorganic NPs, the LOD appeared to decrease (i.e., the sensitivity of LIBD increased) with increasing particle density. It must be noted that particle density is not the sole factor; ionization energy (higher for zeolite compared to polystyrene, titania, and hematite) and light absorption capacity (e.g., stronger light attenuation in the colored hematite) could contribute to sensitivity. The LODs for environmentally relevant PS, PP and PET nanoplastics (with particle densities assumed to be between 0.9–1.4 g mL^−1^) are even one to two orders of magnitude lower than that of PS standards (1 × 10^4^ − 3 × 10^5^ particles mL^−1^ for nanoplastics versus 2 × 10^6^ particles mL^−1^ for PS 100 nm standards). The reason for this improved sensitivity has not been quantitatively examined. The LOD of PS nanoplastics is the lowest, followed by PET and PP nanoplastics.

In summary, LIBD is capable of detecting a variety of hard NPs in clean water, including environmentally relevant nanoplastics, at concentrations relevant to the occurrences of nanoplastics in surface water (10^6^–10^9^ particles mL^−1^). Because of the low LODs toward varied NP types, large detectable concentration ranges (spanning 3−4 orders of magnitude), and in‐line capability, LIBD can be applied in membrane technology for in situ monitoring of permeate quality and membrane integrity. Additionally, LIBD allows further fundamental investigation of membrane mechanisms, such as retention, fouling, breakthrough of probe NPs, and uptake – release of NPs/nanoplastics, via coupling to either the permeate or concentrate streams of the filtration system. Processes where particle aggregation occurs need to be treated with care as aggregation can reduce the breakdown efficiency. Inevitably, this would involve fouling and require solid recovery studies.

In the current research, the effect of complex water chemistry on the sensitivity of LIBD has not been examined, which warrants further investigation. Generally, LIBD is much more sensitive toward hard NPs even though some positive acoustic signals can result from the irradiation of soft colloids at high concentrations (≥3−10 mg L^−1^ for PSS polymers in this work). Further investigation is required to quantify the interference of soft colloids on the detection of hard NPs in mixtures. For multi‐modal solutions of NPs, LIBD can be combined with a size separation method, such as size exclusion chromatography or flow field‐flow fractionation, to simplify the water composition for quantitative analysis. Nanoparticles interact and aggregate in the water environment, and naturally, particle analysis that accounts for such phenomena is and remains a complex challenge.

## 4 Experimental Section

### LIBD System

**Figure 10:**
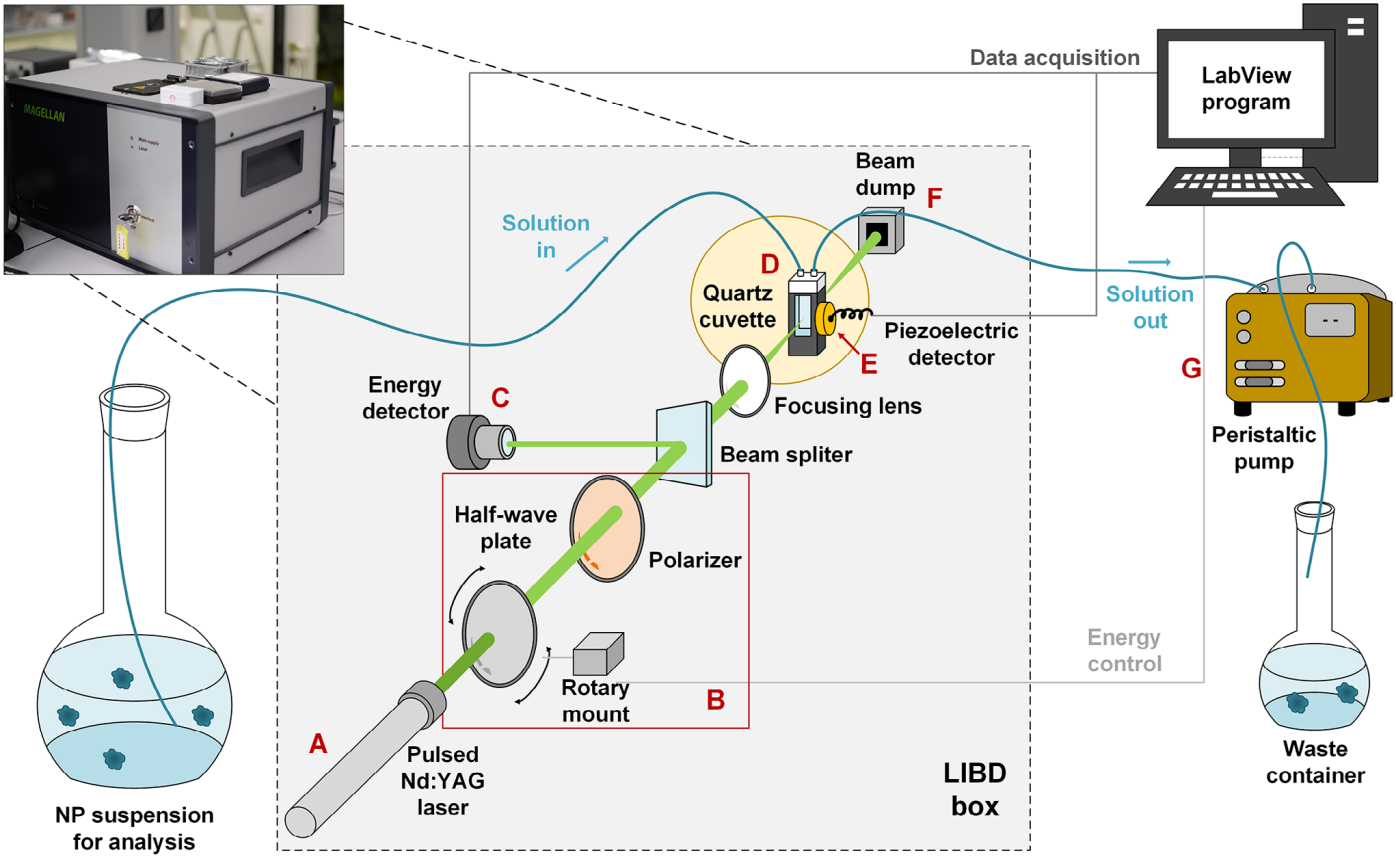
A simplified illustration of LIBD–filtration system. A) laser, B) attenuator system, C) pyroelectric system for laser energy determination, D) sample cuvette, E) piezoelectric detector for BDP determination, F) beam dump, and G) peristaltic pump to induce flow.

The mobile acoustic LIBD system is a prototype system purchased from Cordouan Technologies, which was adapted from a larger system described by Bundschuh et al.^[^ ^54^ ^]^ The mobile system is schematically shown in **Figure** **10**. The light source **(A)** is a pulsed neodymium‐doped yttrium aluminum garnet (Nd:YAG) laser (Flare NX 515‐0.6‐2, Coherent, France) operated at a wavelength of 515 nm (green color). Compared to the original laser,^[^ ^54^ ^]^ the Flare NX laser affords much lower maximum energy (300 µJ, vs 160000 µJ of the original laser – the latter was operated up to only 2200 µJ), but with a much higher pulse frequency (adjustable by dedicated software and as high as 2000 Hz, vs fixed 20 Hz of the original laser), and shorter pulse length (1.3 ± 0.2 ns vs 12 ns of the original laser). The higher pulse frequency may allow faster measurements for in situ monitoring (for example, 3000 laser pulses can be induced within a time as short as 1.5 s). The maximum laser pulse energy in measurements was capped at ≈240−245 µJ, which is low enough to not trigger significant thermal emission (at laser pulse energy above 600 µJ for 100 nm NPs^[^ ^70^ ^]^); hence in all analyses, the BDP was likely limited by the number of electrons released from MPI.

The laser pulse energy was varied between 0 and 240 µJ with an attenuator system **(B)** containing a half‐wave plate attached to a rotary mount (CONEX‐AG‐PR100P, Newport, USA), and a Glan polarizer. A beam splitter directed a portion of the beam for energy determination with a pyroelectric detector **(C)** (QE8SP‐B‐MT‐D0, GenTec EO, Canada). The laser beam was focused (through a focusing lens) into the center of a flow‐through quartz cuvette typically used in UV−vis spectroscopy **(D)**, with an optical path length of 1 cm and volume of 0.75 mL (product ID 175.000‐QS, Hellma Analytics, Germany). The beam diameter at the focus was less than 10 µm, which allowed for the irradiation of multiple NPs (20–400 nm in size) in the focal area. A piezo crystal (custom‐made, Dittel Messtechnik, Germany) detected the breakdowns acoustically **(E)**. The laser beam exiting the cuvette was captured in a laser beam dump (PL15, Newport) **(F)**. The laser pulse energy was controlled and the BDP was recorded by a LabView 2012 program (National Instruments, USA).

In all measurements with varied NP types and concentrations, a fixed flow of 1 mL min^−1^ was induced by a peristaltic pump **(G)** (IPC‐4, Ismatec, Germany, or MiniPlus 2, Gilson, USA). The flow allows the instrument to be evaluated for inline analysis (e.g., when LIBD is connected to a filtration system to determine NP retention in situ). A waste container was connected to the outlet of the peristaltic pump, to collect the sample post‐analysis.

In all analyses, the number of laser pulses per measurement was fixed at 3000, and the frequency of laser pulses was set at 150 Hz (i.e., each measurement lasted 20 s). In analyses with varied laser pulse energies, the laser pulse energy was initially set at 0 µJ (where there are no breakdowns) and increased to 240 µJ in steps of 3−5 µJ with the LabView program. In the calibration at a fixed laser pulse energy, the laser pulse energy was set at the highest value (240 µJ) and the energy step was set at 0 µJ. The NP concentration was exponentially reduced from an initial mass concentration of 1−10 mg L^−1^ by 3.0−3.3 times per step until the LOD was reached (i.e., when there were no “positive” acoustic signals).^[^ ^54^, ^86^ ^]^ A positive signal was recorded if the piezo signal was higher than the pre‐defined threshold, which is a piezo value above the background noise (Figure S1, Supporting Information). The choice of this threshold is important for the separation of the breakdown signals from the background (Figure S2, Supporting Information). The BDP is recorded as the ratio of the number of positive signals to the total laser pulses (3000) – the resolution of BDP is 0.00033. The calculation of BDP is automated with an Excel 2016 macro (copyright of TZW, Germany).

The detailed analytical protocol is described in Table S3 (Supporting Information). With the pump flow rate set at 1 mL min^−1^, the residence time in the cuvette was 45 s. Varying the flow rate between 0 and 4 mL min^−1^, which means varying the residence time between infinity and 11.25 s, affected the LIBD results minimally (Figure S6, Supporting Information).

### Properties of Nanoparticles

A number of NPs used in this study are described in **Table** **1**. Spherical PS standards (density of 1.05 g mL^−1^ and IE of 7.8 eV) with sizes varying between 20 and 400 nm were supplied by Applied Microspheres, Netherlands, as suspensions with a volume ratio of 1% (equivalent to a mass concentration of about 10 g L^−1^). The soft NPs were polystyrene sulfonate (PSS) standards (supplied as powders and flakes, PSS‐Polymer, Germany), with the density (i.e., the mass of polymer molecule divided by its occupied volume in the water matrix) in the order of 0.006 g mL^−1^ and two hydrodynamic sizes of 41 and 142 nm.

**Table 1:** Sizes, densities, and ionization energies of the NPs investigated.

Nanoparticles & Colloids	Source	Sizes [nm]	Density [g mL^−1^]	Ionization Energy IE [eV]
Polystyrene (PS) standards	Applied Microspheres, Netherlands	20, 50, 60, 85, 100, 150, 200, 300, 400^a)^	1.05^a)^	7.8^[^ ^87^ ^]^
Polystyrene sulfonate (PSS) polymer standards	PSS‐Polymer, Germany	41, 142^d)^	0.006^b)^	7.8^[^ ^87^ ^]^
PS nanoplastics	Self‐fabricated^[^ ^34^ ^]^	118^c)^	1.04−1.07^[^ ^88^ ^]^	7.8^[^ ^87^ ^]^
Polypropylene (PP) nanoplastics	Self‐fabricated^[^ ^34^ ^]^	95^c)^	0.87−0.92^[^ ^88^ ^]^	8.6−8.7^[^ ^89^ ^]^
Polyethylene terephthalate (PET) nanoplastics	Self‐fabricated^[^ ^34^ ^]^	100^c)^	1.41^[^ ^88^ ^]^	7.3−7.4^[^ ^89^ ^]^
Porous zeolite FeCZB 30 (SiO_2_/Al_2_O_3_)	Clariant, Switzerland	400^a)^	0.2−0.8 (porous)^a)^; 2.66–2.71 (intrinsic)^[^ ^67^ ^]^	SiO_2_: 12−13^[^ ^90^, ^91^ ^]^; Al_2_O_3_: 8.9^[^ ^67^ ^]^
Rutile Titania (TiO_2_) DiagNano	CD Bioparticles, USA	300^a)^	4.23−4.26 (intrinsic)^[^ ^67^, ^92^ ^]^	7.8^[^ ^93^ ^]^
Hematite (Fe_2_O_3_)	Self‐fabricated^[^ ^94^ ^]^	70 ± 20^[^ ^94^ ^]^	5.24 (intrinsic)^[^ ^67^ ^]^	7.9^[^ ^67^ ^]^

^a)^ Information provided by the manufacturers;

^b)^ Determined from the MWs of 140 and 976 kDa polymers, see Equation (2);

^c)^ Calculated by dividing the mass (MW) of the PSS by the volume that it occupies in the bakground solution, see Equation (3);

^d)^ Modal size determined from NTA analyses.

Environmentally relevant PS, polypropylene (PP), and polyethylene terephthalate (PET) nanoplastics were self‐fabricated from single‐use plastic products based on the protocol published by Rubin et al.^[^ ^34^ ^]^ Single‐use clear teaspoons were used for the top‐down synthesis of PS nanoplastics, whereas large clear pipette tips (1−10 mL) and clear soap bottles were used for the synthesis of PP and PET nanoplastics. Each product was cut into smaller fragments and frozen overnight at −80 °C. The samples were then crushed, milled, and sieved using a stainless‐steel mesh strainer (<100 µm). These particles were used as the raw material for all testing and processes in this study. The raw plastic powders underwent varied accelerated weathering conditions including thermal treatment at 70 °C in an oven (Carbolite, MRC, China), UV irradiation for 5 h in a UV chamber (ProCleaner, BioForce Nanosciences, USA), and probe sonication (QSonica, LLC Q 125, USA) in water suspension on an ice bath for 30 min. Then, the suspension was centrifuged for 10 min at 5000 G to remove large particles, and gradually filtered three times using a 30 µm nylon filter (Whatman, UK), 5 µm nylon filter (Whatman), and finally 0.22 µm PVDF filter (Millipore, USA) in a syringe filter holder (Cole‐Parmer, USA). The permeate of the last filtration step contained suspended nanoplastics with sizes of ∼100 nm and particle counts between 10^8^ and 10^10^ particles mL^−1^, measured with NTA. Unlike the polystyrene standard NPs, the engineered nanoplastics were non‐spherical and had a rough texture; a diverse range of shapes and sizes was found in the suspension of each nanoplastic type.

The metal oxide NPs used include aluminosilicate porous zeolite (SiO_2_/Al_2_O_3_), titania (TiO_2_), and hematite (Fe_2_O_3_). Zeolite FeCZB 30 in powder form with a Si/Al ratio of 25–150 and an average size of 400 nm was purchased from Clariant, Switzerland; due to the porous nature, the particle density varied between 0.2 and 0.8 g mL^−1^, much lower than the intrinsic density of SiO_2_/Al_2_O_3_ (2.66–2.71 g mL^−1^). Titania standards in powder form (rutile DiagNano) were purchased from CD Bioparticles, USA, with a nominal particle size of 300 nm and particle density of 4.23−4.26 g mL^−1^, assuming that the NPs are non‐porous. Hematite NPs were self‐prepared from ferric chloride (FeCl_3_.6H_2_O, ≥99%, Fisher Scientific, Germany) following a published protocol. ^[^ ^95^, ^96^ ^]^ In brief, ferric chloride was hydrolyzed in 3.75 mM HCl (diluted from 37% HCl, Merck, Germany) at 100 °C for 24 h. Subsequently, 0.1 M potassium chloride was added (dissolved from 99.5% powders, VWR, Germany) to initiate aggregation and formation of hematite NPs. The size of hematite NPs obtained was 70 ± 20 nm.^[^ ^94^ ^]^ The prepared stock suspension of hematite NPs (with a mass concentration of 730 mg L^−1^, equivalent to particle number 7.8 × 10^11^ particles mL^−1^) was stored in a cool room (4 °C) at pH 3 to minimize aggregation. The particle density of hematite is 5.24 g mL^−1^, assuming that these NPs were non‐porous.

### Solution Chemistry

Background electrolytes were used in all measurements as a buffer to control the pH at 8.2 ± 0.1 while mimicking the solution chemistry of natural water. The background solution in all experiments contains 1 mM NaHCO_3_ (dissolved from ≥99.7% powders, ENSURE grade, Merck Millipore, USA) and 10 mM NaCl (dissolved from ≥99.9% powders, CHROMANORM grade, VWR Prolabo, Germany) in Milli‐Q water. The high‐quality salts were used because impurities in the background solution may interfere with the breakdown signals of NPs and should be kept minimum.

Polystyrene (PS) standard solutions at 100 mg L^−1^ were prepared by dilution of supplied PS standard suspensions at 10 g L^−1^) 100 times in background electrolytes. Before sample preparation, the supplied suspensions were sonicated in an ultrasonic bath (Elmasonic P, Elma Schmidbauer, Germany, set at frequency 37 kHz and power 70%). The temperature of the bath was set at 21 °C; the actual temperature could rise up to 25 °C. Different concentrations from 3 ng L^−1^ to 30 mg L^−1^ were prepared by diluting these standard solutions in background electrolytes.

Polystyrene sulfonate (PSS) stock solution of 100 mg L^−1^ was prepared by dissolving 100 mg standard powder (PSS‐Polymer, Germany) in 1 L of background electrolytes. Different concentrations were prepared by diluting this stock solution in background electrolytes. The stock suspension of TiO_2_ suspension (with a mass concentration of 2 mg L^−1^ and corresponding particle number ≈1.4×10^7^ particles mL^−1^) was prepared by dissolving 1 mg TiO_2_ powder (CD Bioparticles, USA) in 500 mL of background electrolytes. A higher concentration of TiO_2_ was not prepared because of the increased risk of aggregation and settling. The stock solution was sonicated in the ultrasonic bath for at least 1 h before sample dilution and analysis with LIBD.

Different concentrations of nanoplastics and hematite NPs were prepared by diluting the stock solutions (in the order of 10^9^ particles mL^−1^ for nanoplastics, and 7.8×10^11^ particles mL^−1^ for hematite NPs) in background electrolytes. The stock solutions were sonicated in the ultrasonic bath for at least 1 h before dilution and analysis with LIBD.

A thermo‐coupled pH meter (SenTix 81 connected to pH/cond 3320 device, WTW, Germany) was used to record the solution pH and temperature. The solution pH was 8.2 ± 0.1, and the temperature varied between 21 and 25 °C (i.e., 23 ± 2 °C).

### Nanoparticle Characterization Techniques

Scanning electron microscopy (SEM) was performed to visualize the shape and morphology of NPs and nanoplastics. Several instrument types were used: a Philips XL‐30 ESEM (F.E.I., USA) for the hematite NPs; and a SUPRA 60VP (Carl Zeiss AG, Germany) for the polystyrene standards, titania, and zeolite NPs. The acceleration voltage was fixed at 5 keV, and the secondary electrons were detected with an Everhart − Thornley Secondary Electron Detector. The samples were prepared by drop‐casting 10−50 µL of their respective suspensions in water (particle numbers of ≈10^9^ − 10^12^ particles mL^−1^) onto carbon tape. Then, the samples were left in air to dry and then coated with a 10 nm layer of conductive gold with a sputter coater (SCD 005, BAL‐TEC, Germany). The samples were then characterized in SEM under magnification levels between 1000 and 80000. The micrographs of the NPs at the same magnification are shown in Figure S11 (Supporting Information).

Dynamic light scattering (DLS) with a Litesizer 500, Anton Paar, Switzerland was used to determine NP size and size distribution. The average sizes were determined according to the standard ISO 20412:2008 method,^[^ ^85^ ^]^ although the advanced mode of the instrument was also used to obtain additional details on the size distribution of NPs and the standard deviation of this distribution. Apart from hematite, all NPs showed good colloidal stability in water within up to 7 h, so only one sonication step was required before the full‐range calibration (Figure S13, Supporting Information). For hematite, LIBD analysis was performed immediately (i.e., in less than 3 min) after the sonication of each calibration sample. The size data were validated by performing DLS measurements with PS size standards multiple times (example data of PS 40–100 nm are shown in Figure S17, Supporting Information).

### Quantification of Polystyrene Standard Nanoparticles

Several alternative techniques were used for sensitivity comparison with the LIBD toward the PS standards (20–400 nm in size). The state‐of‐the‐art NTA instrument (NanoSight NS300) was supplied by Malvern Panalytical, UK, equipped with a blue (wavelength 488 nm) laser and a syringe pump. Due to the limitation of the tracking technology, NTA requires NPs to occur at trackable concentrations (at least 10^7^ particles mL^−1^); but the concentration must not be too high to separate individual NPs (maximum 10^9^ particles mL^−1^).^[^ ^41^ ^]^ Hence, the PS standards were prepared to concentrations within this narrow range. Each sample was measured in triplicate of 60 s each in continuous flow mode. Results show that NTA could accurately determine the sizes of PS size standards in the range of 50−300 nm, whereas for 400 nm standards, the measured size was underestimated (Figure S17, Supporting Information).

UV–vis spectroscopy and turbidity tests were used to quantify PS standards in simple matrices (containing only the standards and background electrolytes). UV–vis spectroscopy was performed with a Lambda 25 instrument, PerkinElmer, USA. The suspension samples were transferred into a quartz cuvette (100‐QS, 3.5 mL volume, Hellma Analytics), and measurements were performed at multiple wavelengths in the range of 200−700 nm. As the aromatic rings of PS can absorb light at UV wavelengths, the absorbance of 254 nm was reported to correlate with the PS concentration. However, light attenuation as it passes through the NP suspension is attributed to not only absorption but also scattering. A more accurate absorbance measurement would require the use of an integrating sphere, in which the absorbance/absorptance was measured from the difference between the amount of incident light and the amounts of both transmitted and scattered light.^[^ ^97^ ^]^ Turbidity tests were performed with a turbidity meter (AL450T‐IR, Aqualytic, Germany) following standard method ISO 7007‐1:2006.^[^ ^98^ ^]^ The light wavelength applied was in near‐infrared range (860 nm), and the amount of light scattered at an angle of 90° was measured. Internal calibration was done with <0.1, 20, 200, and 800 NTU standards (provided by the same company Aqualytic).

A TOC analyzer (TOC‐L, Shimadzu, Japan) was used to verify the mass concentration (in mgC L^−1^) of PS standards. The instrument was operated in non‐purgeable organic carbon (NPOC) mode. In brief, the sample was acidified by ortho‐phosphoric acid 25% to convert the inorganic carbon compounds to carbon dioxide gas; then, the carbon dioxide was sparged from the sample suspension. The polymer‐based NPs were not purged and were then subjected to combustion‐catalytic oxidation at 680 °C with platinum catalysts to form carbon dioxide, which was quantified with a non‐dispersive infrared detector. The TOC analysis protocol for the organic matter was applied for the carbon mass concentrations between 0.2 and 10 mgC L^−1^ (the calibration was done with a TOC standard − potassium hydrogen phthalate, dissolved from ≥99.5% powders, Merck Millipore, see Figure S18A). The same TOC analysis protocol for organic matter allows for achieving ≈100% conversion efficiency of the standard NPs (Figure S18B). This is consistent with another report, in which the conversion efficiency of PS 500 nm NPs was as high as 89%.^[^ ^99^ ^]^

### Determination of Particle Count and Particle Density

The particle count of spherical NPs (*n_NP_*, in particles, mL^−1^) of known diameter can be calculated by dividing the mass concentration of the NPs (*c_NP_*, in mg L^−1^) by the particle density ρ_*NP*_ (g mL^−1^) and particle volume, see Equation (1).

(1) $$n_{NP}=\frac{c_{NP}}{\rho_{NP}V_{NP}}=\frac{6\;c_{NP}}{\;\rho_{NP}\;{d_{NP}}^{3}}\;$$

where *d_NP_* (nm) is the NP diameter, and $V_{NP}=\frac{4}{3}\;\pi {\left(\frac{d_{NP}}{2}\right)}^{3}$ (nm^3^) is the NP volume. For PS standards, the particle count calculated from the mass concentration (measured with the TOC analyzer) is consistent with that determined by a particle counting technique (NTA), see Figure S19 (Supporting Information). The calculations for non‐spherical NPs (e.g., nanoplastics and inorganic nanoparticles) assume values for sphere‐equivalents, which means the conversion will not be accurate for titania (polyhedral shapes) and zeolite (distorted porous spheres).

The hydrodynamic sizes of PSS polymers could not be measured due to the large voids between the polymer segments (i.e., low density) resulting in insufficient light scattering. An empirical relationship between the molecular weight (MW, in Da) and hydrodynamic diameter (in nm) is given in Equation (2)^[^ ^100^ ^]^ to estimate the “density” and particle count in the background solution (containing 10 mM NaCl).

(2) $$d_{NP}=\alpha \;\;MW^{\beta }=0.0206\;MW^{\;0.64}$$

where α and β are empirical coefficients. From various works, β varied between 0.57 and 0.70 in the presence of different NaCl concentrations (between 0 and 500 mM), whereas α varies between 0.014 and 0.021.^[^ ^100^, ^101^, ^102^ ^]^ The polymer “density” (analog to the particle density for hard NPs) is then calculated following Equation (3).

(3) $$\rho_{NP}=\frac{\mathrm{MW}}{N_{Avo}\;V_{NP}}=\frac{6\;\mathrm{MW}}{\pi \;N_{Avo}\;{d_{NP}}^{3}}\;$$

where *N_Avo_* is the Avogadro number. For PSS, ρ_*NP*_ ≈ 0.006 g mL^−1^ (i.e., 6 mg of PSS solid occupies a volume of 1 mL in the background solution). The count of PSS polymers was determined via Equation (1).

### Error Analysis

The error analysis procedure is described in Table S4 (Supporting Information). The main error sources include solution preparation with pipette and volumetric flask (negligible variations), solution temperature and chemistry (small variations), and LIBD analytical error. The determination of the analytical error (as standard deviations) from multiple measurement repeats is illustrated in Figures S3 and S4 (Supporting Information). The analytical error contributed the most to the uncertainty in LIBD results; the lower the BDP, the higher its relative error. For example, the relative error is 1% when BDP = 0.92, 8% when BDP = 0.26, and 13% when BDP = 0.06 (Figure S4, Supporting Information). At the LOD, the relative error is as high as 50% (Figure S5, Supporting Information).

## Conflict of Interest

The authors declare no conflict of interest.

## Supporting information



Supporting Information

## Data Availability

The data that support the findings of this study are available from the corresponding author upon reasonable request.
