# Supplementary material for: Quantification of Nanoplastics and Inorganic Nanoparticles via Laser‐Induced Breakdown Detection (LIBD)
Source: Small Methods. 2025 Apr 8;9(9):2402060. doi: 10.1002/smtd.202402060 (PMC12464807; doi:10.1002/smtd.202402060)
Supplement: Supplementary file 1 — Supporting Information [file SMTD-9-2402060-s001.pdf]

small
methods

Supporting Information

for *Small Methods*, DOI 10.1002/smtd.202402060

Quantification of Nanoplastics and Inorganic Nanoparticles via Laser-Induced Breakdown Detection (LIBD)

*Minh N. Nguyen, Pia Lipp, Ines Zucker and Andrea I. Schäfer**

Supporting Information

Quantification of inorganic nanoparticles and nanoplastics via laser-induced breakdown detection (LIBD)

Minh N. Nguyen [1], Pia Lipp [2], Ines Zucker [3], Andrea I. Schäfer [1]*

[1] *Institute for Advanced Membrane Technology (IAMT), Karlsruhe Institute of Technology (KIT), Hermann-von-Helmholtz-Platz 1, 76344 Eggenstein-Leopoldshafen, Germany*

[2] *TZW: DVGW-Technologiezentrum Wasser, Karlsruher Str. 84, Karlsruhe 76139, Germany*

[3] *School of Mechanical Engineering, Faculty of Engineering, Tel Aviv University, Tel Aviv 69978, Israel*

1. Laser-induced breakdown detection (LIBD) operation

1.1. Registration of breakdown acoustic signals

To determine the breakdown probability (BDP) from LIBD measurements, it is important to distinguish the acoustic signals resulting from breakdowns from the background noise. A signal threshold is set by the operator; when the piezo signal is higher than this threshold, it is registered as a positive result; the higher number per 3000 laser pulses, the higher the BDP. Examples are given in **Figure S1** for a polystyrene (PS) 20 nm standard nanoparticles (NPs) with three pulse energies of 22, 81 and 120 µJ. 1000 measurements were made at each pulse energy. The threshold is set at 1.0.

At a low pulse energy (22 µJ), there was no breakdown as all the piezo signals were below the 1.0 threshold. At a higher pulse energy (81 µJ), there was around 170 measurements with piezo signals higher than the threshold, indicating that a proportion of laser pulses caused breakdowns (BDP = 0.17). At an even higher pulse energy (120 µJ), breakdowns were detected in nearly 60% of measurements, hence BDP = 0.6.

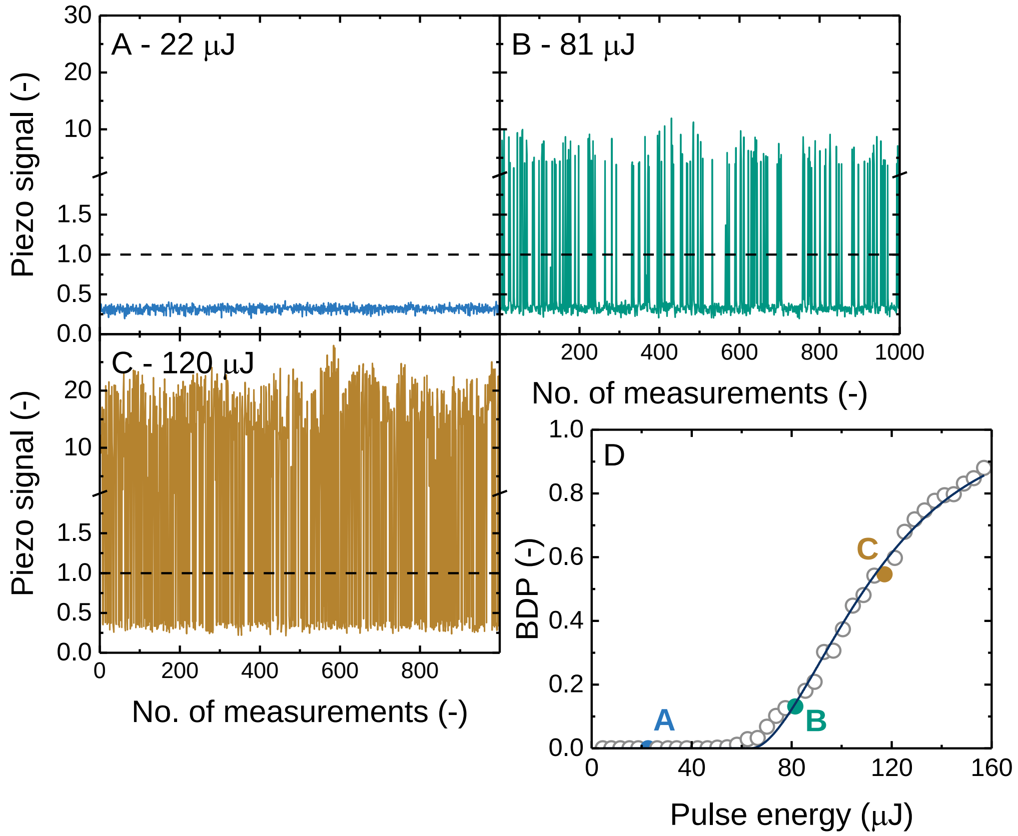

Figure S1. A, B and C – Piezo signals obtained in 1000 measurements with each laser pulse energy (22, 81 and 120 μJ, respectively) for PS 20 nm standard NPs at 1 mg L^{-1}. D – BDP (determined as the number of breakdowns detected per 1000 measurements) at different laser pulse energies.

1.2. Mathematical determination of acoustic signal threshold

Because selecting a too high or too low signal/noise threshold can result in under- or overestimation of BDP, respectively (as signals are mistaken as background noise and *vice versa*), a plausible methodology to calculate the threshold is given as follows.

Step 1: Determine the average $\bar{x}$ and standard deviation σ_x of the background noise (conditions: only Milli-Q water or pure background electrolytes, at very low pulse energy *e.g.* 10 μJ).

Step 2: Calculate the signal threshold x_{th} (*i.e.* limit of quantification) as the average background signal multiplied by ten times the background signal standard deviation. This threshold ensures that the background noise is not mistaken as the signal [1].

$$x_{th} = \bar{x} + 10\,\sigma_x \tag{1}$$

Step 3: Analyze the piezo signal data with this threshold value.

To visualize how BDP values can be impacted by the choice of signal threshold, **Figure S2** shows the BDP vs. pulse energy of a particular data set (PS 50 nm standards at 1 mg L^{-1} concentration),

analyzed with a range of signal thresholds between 0.1 and 15. The threshold calculated with Eq. (1) is 0.7.

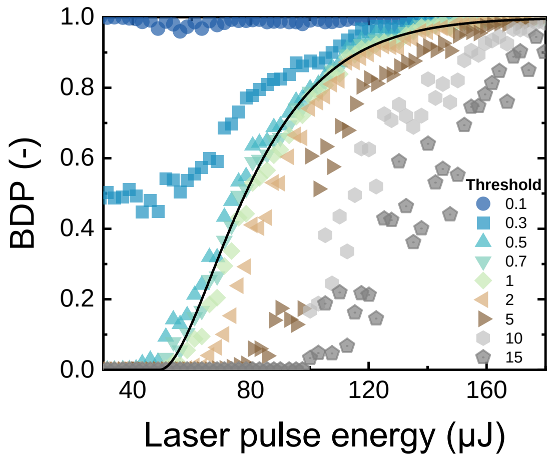

Figure S2. BDP vs. laser pulse energy for PS 50 nm standards at 1 mg L^{-1}, analysed with varying signal thresholds. The black curve is the S-curve fit with the threshold 0.7 data. Flow rate 1 mL min^{-1}, 1 mM $NaHCO_3$, 10 mM NaCl, pH 8.2 ± 0.1, 23 ± 2 °C.

With the thresholds set at 0.1 and 0.3, the BDP was overestimated and an S-curve could not be observed because the background noise was mistaken as breakdown signals. By setting the threshold between 0.5 and 1, a uniform S-curve could be obtained. With thresholds set above 10, the S-curve were shifted to the right because some early breakdown could not be detected, and lost its shape because many breakdown signals were mistaken as noise.

1.3. Intrinsic analytical error of the LIBD

To examine whether the BDP measured at the same laser pulse energy with the same NP type and concentration followed a normal distribution, the BDP from 250 repeats was reported in **Figure S3**.

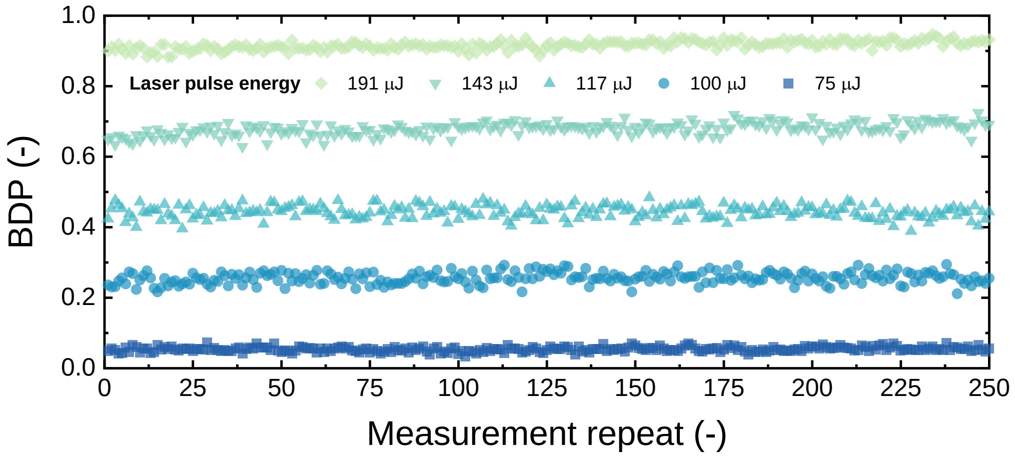

Figure S3. BDP measured 250 times at five laser pulse energies (± 3 µJ). PS particle size 20 nm, concentration 1 mg L^{-1} (or $2.3 \cdot 10^{11}$ particles mL^{-1}). Dashed horizontal lines indicate the average BDP. Flow rate 1 mL min^{-1}, 1 mM $NaHCO_3$, 10 mM NaCl, pH 8.2 ± 0.1, 23 ± 2 °C.

For the analysis of a single suspension, BDP was higher at a higher laser pulse energy (from 75 to 191 µJ). At the same laser pulse energy, BDP appeared to vary randomly between the 250 repeats. The number of repeats was chosen to ensure that a random (Gaussian) distribution was clearly observed. The frequency distribution was then plotted in **Figure S4** to determine the standard deviations.

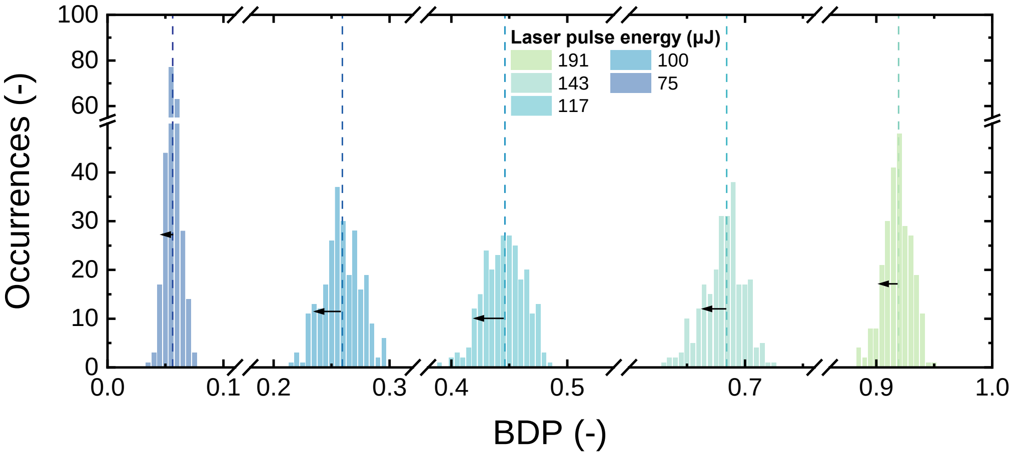

Figure S4. Histogram frequency from data of 250 repeats per laser pulse energy (± 3 µJ). PS particle size 20 nm, concentration 1 mg L^{-1} (or $2.3 \cdot 10^{11}$ particles mL^{-1}). The Gaussian fits are included. Flow rate 1 mL min^{-1}, 1 mM $NaHCO_3$, 10 mM NaCl, pH 8.2 ± 0.1, 23 ± 2 °C.

A random (Gaussian) distribution of BDP was achieved at each specific pulse energy. The average BDPs ± standard deviations are 0.92 ± 0.01 (1%), 0.68 ± 0.02 (3%), 0.44 ± 0.02 (5%), 0.26 ± 0.02 (8%) and 0.053 ± 0.068 (13%) at laser pulse energies of 191, 143, 117, 100 and 75 µJ, respectively. These values may vary for other particle types and concentrations. It appears that the higher BDP, the lower the relative error.

In summary, similar error ranges will be obtained from 40 measurements (in a ~ 12 min analysis) instead of 250 (requiring an analytical time of 83 min). Hence for concentration error analysis in LIBD calibration, a reduced number of repeats of 40 was chosen to reduce analysis time.

1.4. Determination of the detection limit of LIBD

The detection limit was selected as the mass concentration or particle count, below which a large percentage of measurements are negative (*i.e.* zero breakdown signals) and the BDP in the remaining measurements no longer followed a Gaussian distribution. The histograms of BDP at mass concentrations of PS 100 nm standards above (1 µg L^{-1}) and below (0.3 µg L^{-1}) the detection limit are shown in **Figure S5**.

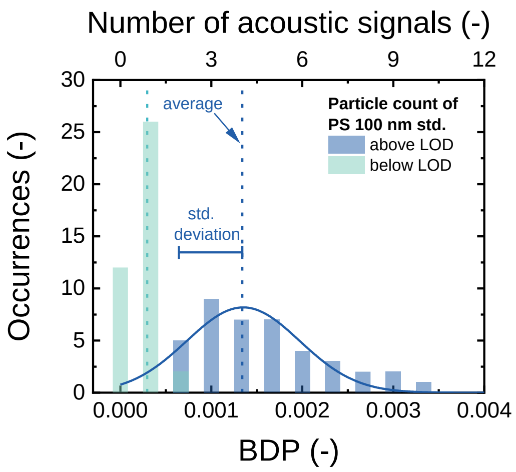

Figure S5. Histograms of BDP and number of acoustic signals (per 3000 laser pulses) at two concentrations of PS 100 nm standard NPs: 1 µg L^{-1} (above the detection limit, blue bars) and 0.3 µg L^{-1} (below the detection limit, green bars). The vertical dotted lines indicate the average BDP. The solid blue curve indicates the Gaussian fit for the BDP at 1 µg L^{-1} concentration. The BDP at 0.3 µg L^{-1} cannot be fitted.

At a mass concentration of 1 µg L^{-1} (above detection limit), the BDP distribution followed a normal distribution. The standard deviation (0.0007) determined from 40 measurements was smaller than the average value (0.0014). The relative error reported as the ratio of the standard deviation to the average was 50%. At a mass concentration of 0.3 µg L^{-1} (below detection limit), a large proportion of measurements was negative (*i.e.* 12 out of 40 measurements registered zero acoustic signals). This means particles at this concentration are likely undetectable. Because BDP = 0 was found in multiple repeats, the 0.3 µg L^{-1} data did not follow a normal distribution. Hence the error bar for data below the LOD is larger than the average value itself.

1.5. Impact of flow rates on LIBD measurements

Flow of solution is necessary when LIBD needs to be coupled with for example a filtration system for online monitoring. To determine if the flow rate affects the BDP, the BDP plus standard deviations at varied flow rates (from 0 to 4 mL min^{-1}) are given in **Figure S6**.

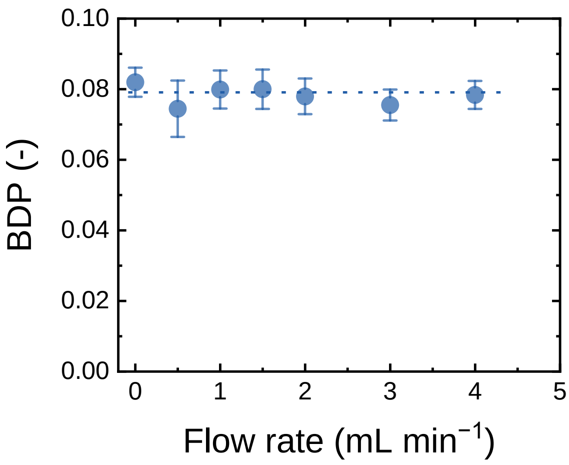

Figure S6. BDP measured at a fix laser pulse energy of 240 µJ vs. flow rate. 100 µg L^{-1} PS 100 nm standards, 1 mM $NaHCO_3$, 10 mM NaCl, pH 8.2 ± 0.1, 23 ± 2 °C. The error bars indicate standard deviations from 40 repeats.

The BDP was found independent of flow rate in the range of 0 – 4 mL min^{-1}.With the flow rate increasing from 0 to 4 mL min^{-1}, the residence time in the cuvette decreases from ∞ to 11 s.

2. Comparison of different techniques for nanoparticle size / concentration determination

Table S1. NP size and number determination techniques, with limits of detection for hard NPs (such as metal oxides and polystyrene NPs). Abbreviations: S – particle size, N – particle number.

Technique	Type	Size LOD (nm)	Number LOD (particles mL^{-1}) + conditions	In-line (Yes / No)	Comments
**Light scattering techniques**					
Dynamic light scattering (DLS)	S	< 1 [2, 3]	N.A.	Yes	
Small-angle X-ray scattering [4, 5]	S	1 [5, 6]	N.A.	Yes [6]	
Nanoparticle tracking analysis (NTA) [7]	S & C	20 − 30 [7, 8]	10^7–10^9 [7, 8]	Yes	
Multi-angle light scattering	S & C	< 1 (same as DLS)	10^{10} (20 nm); 10^8 (≥ 100 nm) [9]	Yes [9]	
Single particle counting (SPC)	S & C	50 – 100 [10]	10^4 (50 – 100 nm); 10^2 (400 – 500 nm) [10]	Yes	
Laser diffraction [11]	S & C	100 [11]	10^9 (200 nm, silica) [12]	Yes	
Electrospray-scanning mobility particle sizer	S & C	1 [13]	$2 \cdot 10^9$ (50 − 125 nm); 10^{11} (5 nm) [13]	Yes	
**Mass spectrometry techniques**					
Single-particle ion-coupled plasma – mass spectrometry (spICP-MS) [14, 15]	S & C	6 – 500 [16]	10^6 [14]	Yes [17]	Applicable for metallic/metal oxide NPs
Pyrolysis − gas chromatography – mass spectrometry (Py-GC-MS) [18, 19]	C	N.A.	0.04 − 0.44 µg/L**	No	Useful for nanoplastics
Matrix-assisted laser desorption/ionization time-of-flight − mass spectrometry (MALDI-TOF-MS)	C	N.A.	25 mg/L** (100 – 200 nm) [20]	Yes	
**Microscopy techniques**					
Scanning electron microscopy (SEM)	S	1 [21]	N.A.*	No	
Transmission electron microscopy (TEM)	S	0.2 [22]	N.A.*	No	
Atomic force microscopy (AFM) [23, 24]	S	4−5 [24]	N.A.*	No	
**Micro-/nanofluidic techniques**					
Resistive pulse sensing (RPS) [25, 26]	S & C	~ 50 [27]	10^9 (100 nm) [28]; 10^8 (400 nm) [29]	Yes	
Resonance mass measurement (RMM) [30]	S & C	~ 200 (for PS) [31]	10^7 (400 and 1000 nm) [31]	No	Sensitivity depends on particle density
**Other techniques**					
Laser-induced breakdown detection (LIBD) [32]	S & C	5 − 20 [33, 34]	$3 \cdot 10^6$ (20 nm); $7 \cdot 10^4$ (500 nm) [34]	Yes	

* Particle concentration can be estimated from the number of particles detected within the field of view. ** Mass concentration is reported instead of particle number.

3. Fitting of the double logarithmic calibration with polystyrene standard particles

To evaluate the linearity of the calibration with polystyrene standard particles, the fitting parameters of the double logarithmic relationship between the BDP and particle concentration (in particles mL^{-1}) is given in **Table S2**.

Table S2. Fitting parameters of the double logarithmic relationship between the BDP (between 0.003 and 0.7) and particle concentration n_{NP} (in particles mL^{-1}): $\log(BDP) = a \log(n_{NP}) + b$.

Particle size	Fitting parameter a	Fitting parameter b	R^2-value of the fit
20	0.979	−9.918	0.989
50	0.804	−7.535	0.994
60	0.875	−8.196	0.992
85	0.866	−8.040	0.997
100	0.902	−8.431	0.996
150	0.957	−8.614	0.998
200	0.902	−7.974	0.997
300	0.842	−7.467	0.984
400	0.839	−7.302	0.996

The fitting parameter a indicates the linearity of the BDP and particle concentration relationship (a = 1 results in BDP $= 10^b n_{NP}$, which means BDP increases linearly with particle concentration in the normal scale). The regression slope on a double logarithmic scale remains consistent at approximately 0.9 ± 0.1 with different PS standard sizes, which indicates a relationship that is nearly linear in the normal scale. The R^2-value of the fit is between 0.984 and 0.998, which means the quality of the fit is relatively high.

4. Calibration of particle counting techniques with polystyrene standard nanoparticles

4.1. UV-Vis spectroscopy

UV-Vis spectrophotometry (which relies on the light absorption and scattering property of NPs) was performed to compare analytical LODs with LIBD (which relies on the ionization capability of the NPs caused by green laser irradiation). Firstly, to examined the absorption of light at varied wavelengths, the UV-Vis absorbance spectra of PS nanoparticles at wavelengths 200 − 700 nm (**Figure S7**).

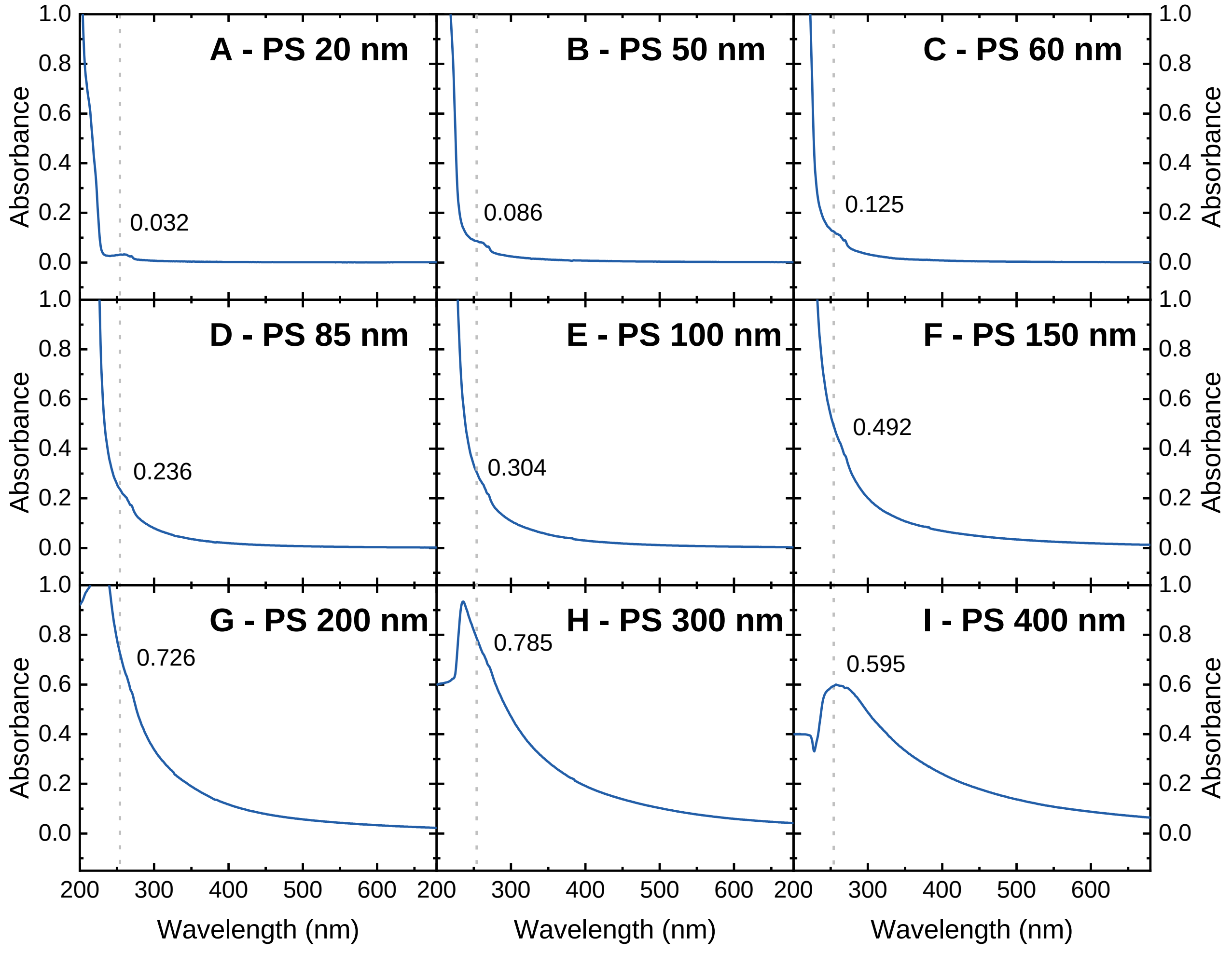

Figure S7. Absorbance at different wavelengths (200 − 700 nm) of PS nanoparticles at a uniform mass concentration of 10 mg L^{-1}. Numbers indicate absorbance at the wavelengths of 254 nm (vertical dotted lines), which will be selected for particle number calibration. 1 mM $NaHCO_3$, 10 mM NaCl, 23 ± 2 °C.

All PS sizes show strong UV absorption and negligible absorption in visible light range. Absorbance (actually the measurements are for photon loss due to both absorption and scattering) at 254 nm generally increases with increasing particle size although the mass concentration is fixed (10 mg L^{-1}).

Subsequently, the calibration (254 nm absorbance with varied concentrations) was plotted against particle count to determine the detection range and limit of detection (LOD) (**Figure S8**).

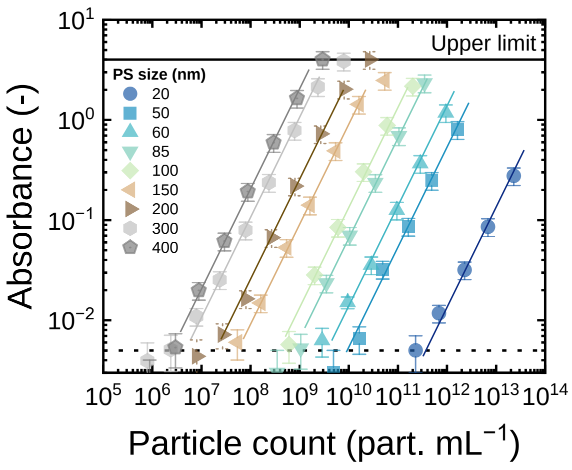

Figure S8. Absorbance at 254 nm vs. particle concentration for PS standard sizes 20, 50, 60, 85, 100, 150, 200, 300 and 400 nm. 1 mM $NaHCO_3$, 10 mM NaCl, 23 ± 2 °C, pH 8.2 ± 0.1.

A double logarithmic regression was found between absorbance and particle concentration. Because absorbance is proportional to concentration with a power factor of 1, the slope of the double logarithmic relationship is 1. Lower particle concentration ranges were achieved for larger particle sizes. The LOD reduced with increasing particle size: ~10^{12} particles mL^{-1} for 20 nm standards, and 10^7 particles mL^{-1} for 400 nm standards.

4.2. Turbidity measurements

Turbidity measurements (which rely on the near-infrared light scattering property of suspended NPs) were performed to compare analytical LODs with LIBD. The calibration (turbidity in NTU vs. particle count) for varied PS standard sizes is shown in **Figure S9**.

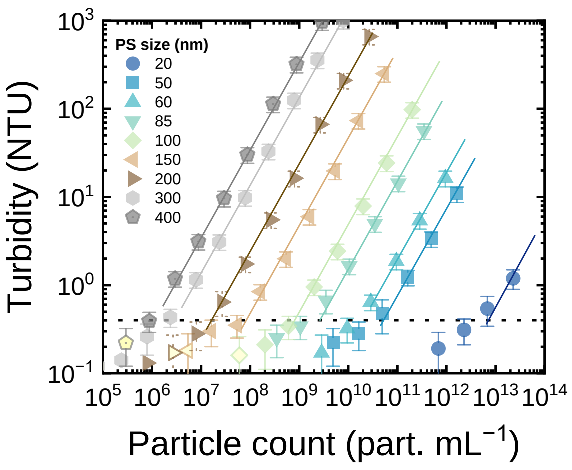

Figure S9. Turbidity vs. particle concentration for PS standard sizes 20, 50, 60, 85, 100, 150, 200, 300 and 400 nm. 1 mM $NaHCO_3$, 10 mM NaCl, 23 ± 2 °C, pH 8.2 ± 0.1.

A double logarithmic regression was found between turbidity and particle count. Because turbidity is proportional to the particle count with a power factor of 1, the slope of the double logarithmic relationship is 1. Lower particle concentration ranges were achieved for larger particle sizes. The LOD reduced with increasing particle size: ~10^{13} particles mL^{-1} for 20 nm standards, and $5 \cdot 10^6$ particles mL^{-1} for 400 nm standards.

4.3. Nanoparticle tracking analysis

Nanoparticle tracking analysis (which relies on the optical detection of individual NPs *via* light scattering of blue laser light) was another alternative technique for particle counting. The calibration (measured particle count vs. expected particle count) is given in **Figure S10**.

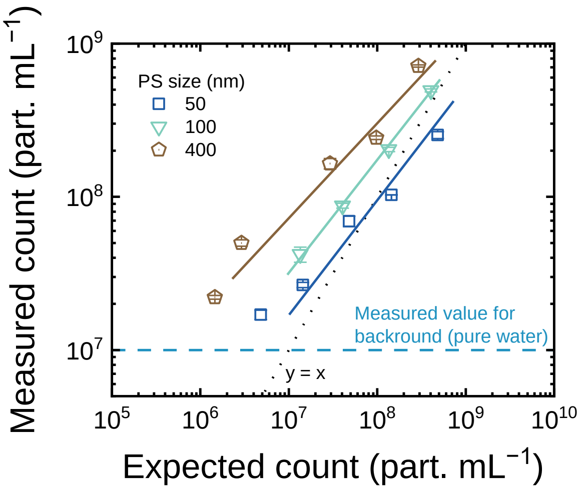

Figure S10. NTA results − measured particle count is plotted against expected particle count for PS standard sizes 50, 100, and 400 nm. 1 mM $NaHCO_3$, 10 mM NaCl.

A double logarithmic regression was found between the measured particle count and the expected particle count, which means NTA can detect different concentrations of NPs within a relatively narrow working range of 10^7 – 10^9 particles mL^{-1}. The NTA measured results were higher than the expected results for both particle sizes 100 and 400 nm; the measured and expected results were consistent for 50 nm standards.

5. Properties of inorganic nanoparticles

5.1. Shape and surface morphology

The nanoparticles (NPs) were visualized with scanning electron microscopy (SEM) to qualitatively determine the particle shapes, morphologies, and sizes. PS standards (100 nm), titania, zeolite and hematite were viewed under the same magnification as shown in **Figure S11**.

The PS 100 nm standards (**Figure S11 A**) and hematite (**Figure S11 D**) show relatively uniform spherical shapes and particle sizes (100 nm and 85 nm, respectively). The spherical shapes can be expected for all the hard PS standards with sizes varying from 20 to 400 nm. In **Figure S11 C**, the pores can be observed in zeolite as little dots on the distorted spheres. The titania NPs in **Figure S11 B** are polyhedral and non-uniform in size.

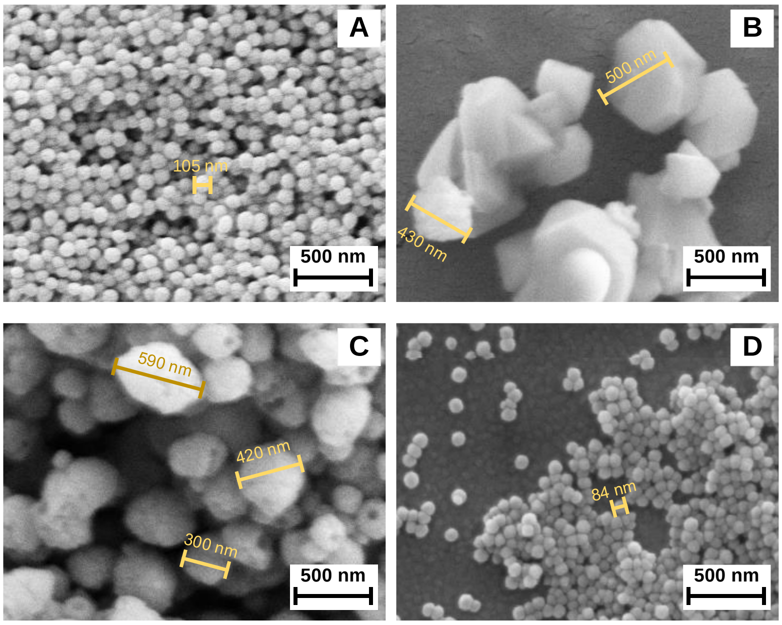

Figure S11. SEM images of various nanoparticle types at the same scale. A – PS 100 nm standards, B – titania, C – zeolite, and D – hematite.

5.2. Hydrodynamic sizes and size distribution

The distribution of the hydrodynamic sizes of inorganic and PS standard NPs, measured within 5 min after sonication, is given in **Figure S12**; this complements the size distribution analysis of nanoplastics shown in the main text. The PSS polymers could not be characterized in DLS due to very low density, as the large void volume results in minimal light scattering.

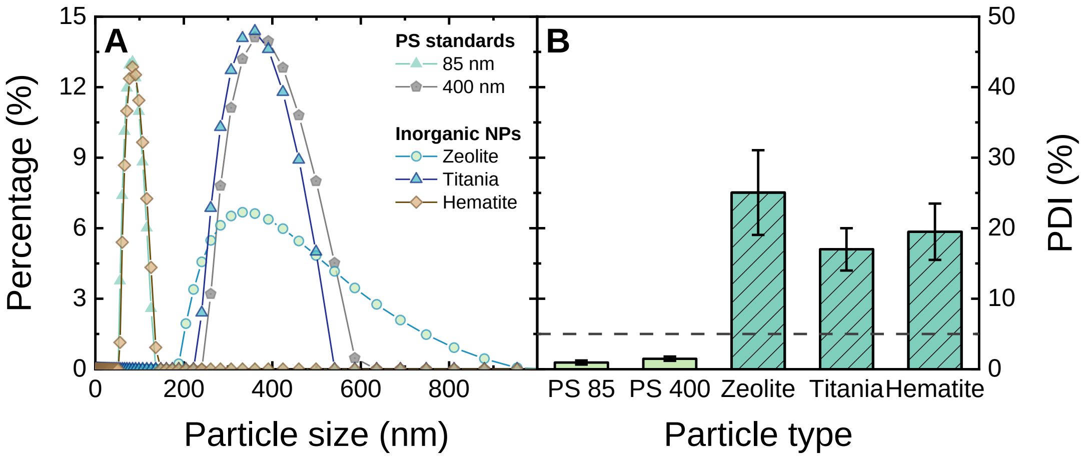

Figure S12. Size distribution (A) and polydispersity index (PDI) (B) of inorganic NPs compared with PS standards (85 and 400 nm), measured with DLS. Horizontal dashed line in B indicate the polydispersity threshold. 1 mM $NaHCO_3$, 10 mM NaCl, 20 °C.

From **Figure S12 A**, the size distribution of hematite NPs is largely similar to that of PS 85 nm standards; while the size distribution of titania and hematite varies around 400 nm; the distribution of sizes of zeolite is larger than that of titania and the PS 400 nm standards. **Figure S12 B** reveals

that all the inorganic NPs are polydispersed (PDI greater than 5 % is an indication of polydispersity [35]) ; this contrasts the PS standards (< 5% PDI), which are known to be monodispersed.

The colloidal stability of NPs, including nanoplastics, will affect the detection of these particles with LIBD. DLS was used to examine the change in nanoparticle sizes from ~1 min to 7 h after ultrasonication was examined in **Figure S13**.

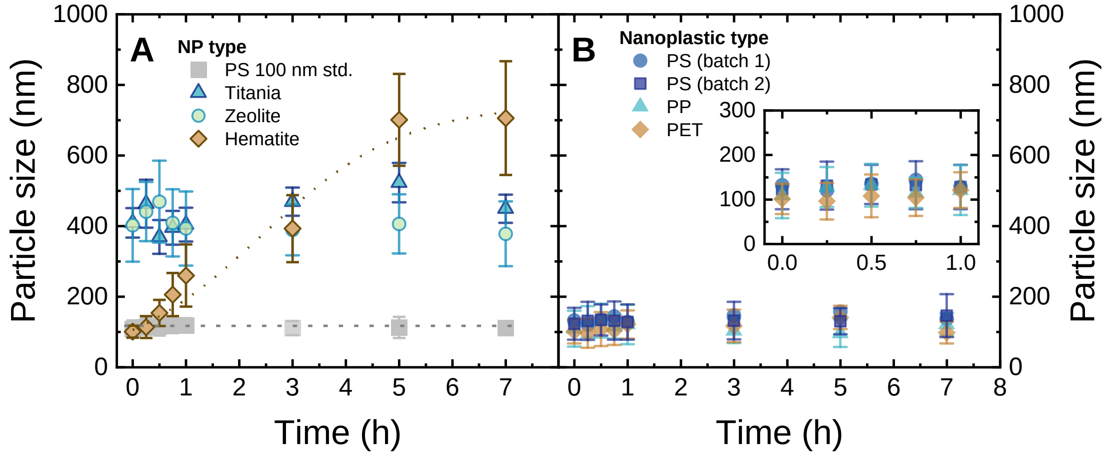

Figure S13. A and B − Changes in size of various NPs over time (following ultrasonication). The inset in B zooms in the size range 0 – 300 nm and time 0 – 1 h, 1 mM $NaHCO_3$, 10 mM NaCl, 20 °C. Error bars indicate the standard deviations of size distribution determined from DLS.

The particle size of PS 100 nm standards and PS nanoplastics did not change significantly over time (between 1 min and 7 h), indicating that the nanoplastics were stable and could be characterized with LIBD in a full-day experiment. Other PS size standards should behave the same as PS 100 nm. Sizes of titania and zeolite NPs were relatively uniform over time at around 400 nm and could also be characterized with LIBD in a full-day experiment. The particle size of hematite increased rapidly over time, from 100 nm at 0 h to 710 nm at 7 h, which indicates strong agglomeration. Within the first 15 min the measured particle size was uniform (100 ± 23 nm); hence, each hematite sample needed to be sonicated and then immediately measured with LIBD (within a 15 min margin).

5.3. Light absorption capability

To determine how much the NPs can absorb laser light (light absorption is a potential factor that affects the BDP), which can be related to the utilization of photon energy for ionisation, the visible light absorption of different NPs is reported in **Figure S14**, at the same total volume of particles (1−2) · 10^{-6} mL mL^{-1}.

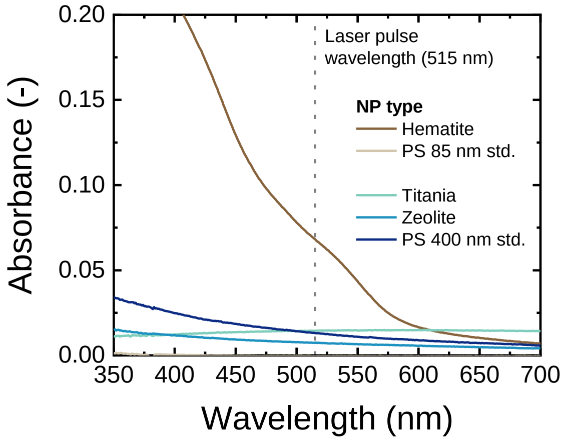

Figure S14. Absorbance at 350 − 700 nm for PS standard sizes 85 and 400 nm. Measured by UV-Vis spectroscopy. Hematite (70 ± 20 nm), titania (~ 400 nm) and zeolite FeCZB 30 (~ 400 nm). 1 mM $NaHCO_3$, 10 mM NaCl, 23 ± 2 °C.

The absorbance of hematite at 515 nm (absorbance = 0.06) is much higher than the PS 85 nm counterpart (absorbance ~ 0), hence more photon energy can be taken up and utilized for ionization. Titania, zeolite and PS 400 nm show only slight variation in absorbance.

6. Breakdown of polystyrene standards at different laser pulse energies

To identify the energy threshold where the first sign of breakdowns was observed for all the PS standard sizes, the BDP was plotted at varied particle concentrations from the orders of 10^{10} or 10^9 to 10^6 particles mL^{-1} for PS standard sizes 50 – 400 nm (**Figure S15**). The curves for PS 20 nm standards are not shown in **Figure S15** but in the main text.

As observed for all the NP sizes, between a laser pulse energy of 0 and 36 µJ, the BDP was equal to zero and no acoustic signal was detected. The energy threshold was within the range of 36 – 60 µJ measured at $10^8 - 10^9$ particles mL^{-1}. The energy threshold appears to decrease as the particle size increases. For instance, the energy thresholds for 50, 100, and 400 nm standard particles are 53, 44, and 37 µJ, respectively. In the laser pulse energy range between 50 and 240 µJ, the BDP increases with increasing laser energy. At the same energy, the higher concentration, the higher BDP.

It is noted that the LIBD is capable of determining the particle size from the laser threshold energy as shown in earlier studies [36, 37]; however, the LIBD prototype used in this work was not applied for particle size determination. Nevertheless, to evaluate this possibility, the laser threshold energy (selected as the energy at which ≥10 breakdown signals were detected) of the LIBD used in this study was compared to that of a pioneering study [37] (**Figure S16 A** and **B**, respectively). The latter system was bulkier and employed a strong pulsed laser (with a higher maximum energy of 10,000 µJ) with a higher laser wavelength (532 nm). Due to significant differences in experimental setup, the threshold energy values between the two systems are not directly comparable, although it is possible to assess the general trends in threshold energy relative to particle size.

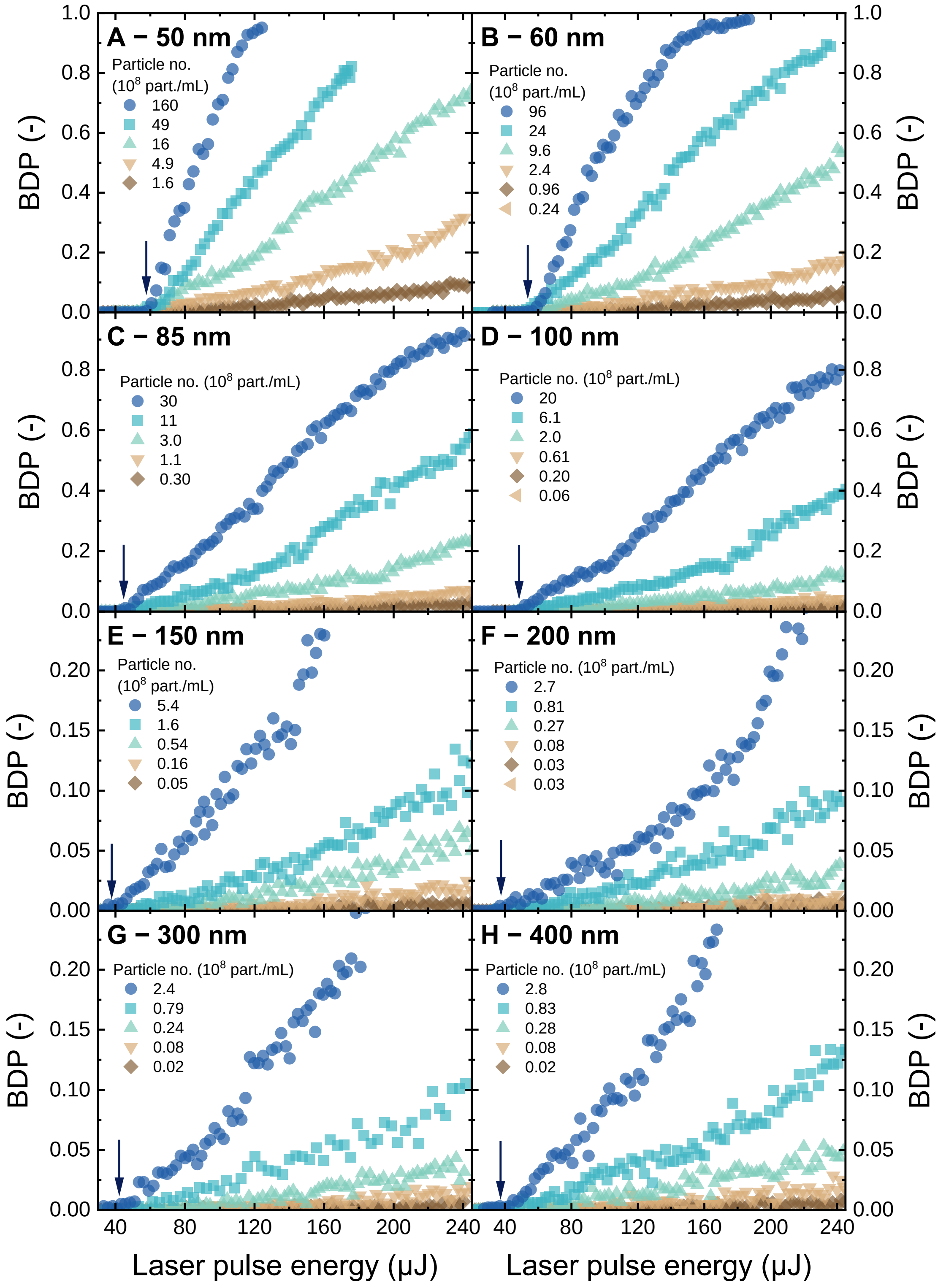

Figure S15. BDP vs. laser pulse energy for PS 50 − 400 nm at different concentrations. Flow rate 1 mL min^{-1}, 1 mM $NaHCO_3$, 10 mM NaCl, 23 ± 2 °C, pH 8.2 ± 0.1. Vertical arrows indicate the positions of the energy thresholds, *i.e.* when the BDP reached ≥ 0.003 implying the minimum energy at which breakdown starts to happen.

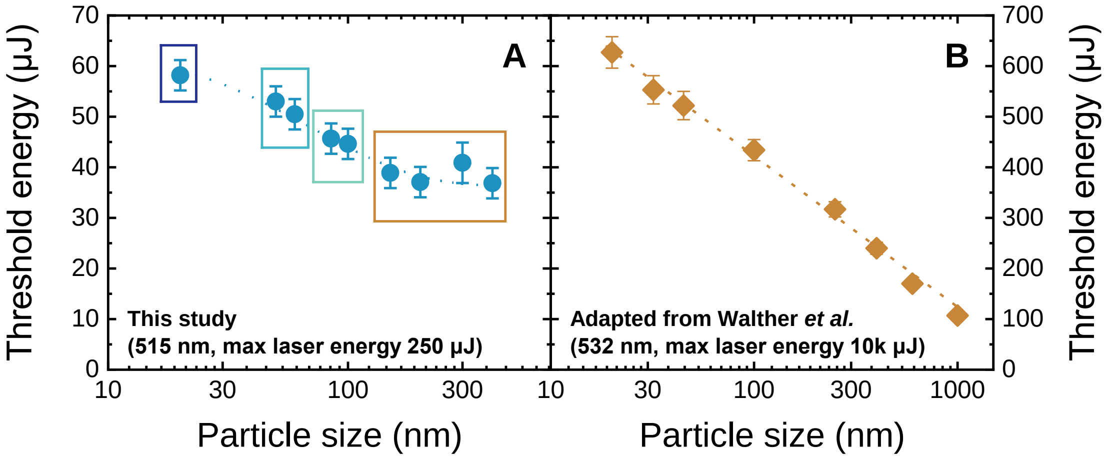

Figure S16. Threshold energy as function of particle size attained with the LIBD systems used in this study (A) and in a previous study on LIBD (graph adapted from Walther *et al.* [37]) (B). The hollow boxes in A indicate the particle size groups with similar laser threshold energies.

From **Figure S16 A**, the threshold energy determined in this study generally decreased with increasing particle size. However, when the particles were close in size, the differences become indistinguishable. Four particle size groups can be identified: 20 nm, 50−60 nm, 85−100 nm, and 150−400 nm, within each of which particles showed comparable threshold energies. It is then challenging to obtain the particle size from the LIBD results of BDP at varied laser pulse energies, and consequently, to accurately determine particle concentration if the particle sizes are unknown.

Figure S16 B suggests that a stronger laser, such as the one used by Walther *et al.* (with a maximum energy of 10,000 µJ), and possibly a higher laser wavelength (532 nm) could improve the differentiation of particle sizes based on threshold energy. Using Walther *et al.*'s set-up, both particle size and concentration can be determined from the S-curve of BDP versus laser pulse energy [37]. Additionally, this set-up allows for estimating the concentration of particles with multiple sizes in a multi-modal suspension [38]."

Materials and Methods

7. Particle size and number determination of polystyrene standards

7.1. Size determination

The particle sizes of polystyrene (PS) standards are confirmed with DLS to dismiss the possibility of aggregate formation which can affect the LIBD calibration results (**Figure S17**). Additionally, the capability of NTA in determining standard particle sizes was examined.

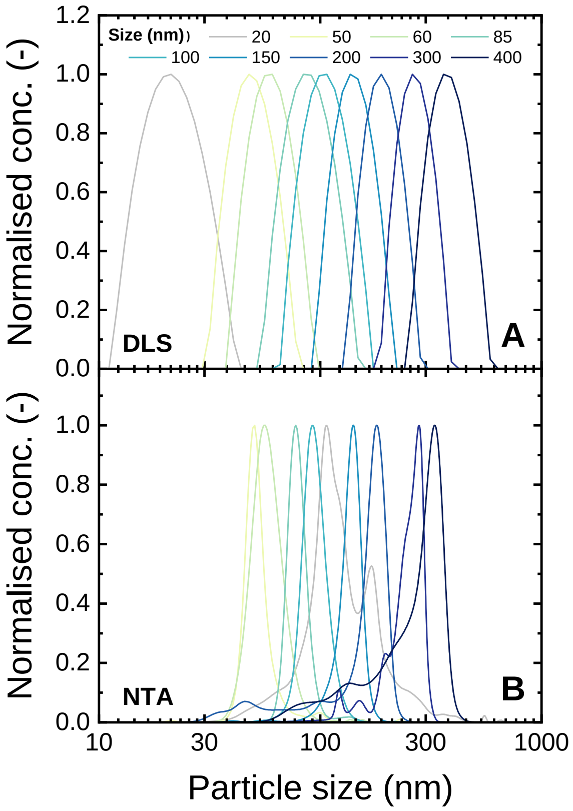

Figure S17. DLS (A) and NTA (B) size results for 20 − 400 nm PS standard particles, given as normalized concentration (particle count at a specific size divided by the peak particle count) vs. particle diameter in log scale. 1 mM $NaHCO_3$, 10 mM NaCl, 23 ± 2 °C, pH 8.2 ± 0.1.

From DLS results (**Figure S17 A**), all the standard solutions were monodispersed (a single peak was observed per PS size). Good agreement was found between the determined particle size in DLS and nominal particle size. For NTA (**Figure S17 B**), the PS 20 nm standards were not correctly detected. The modal size of PS 400 nm was also underestimated (323 nm), and from 150 to 400 nm, some interference caused by the smaller-sized particles was detected.

However, the widths of the peaks in NTA are smaller than those in DLS, which may mean that NTA provided more precise size results than DLS. In summary, the DLS and NTA methods can both be validated with the standard PS (noting that the concentration range of NTA was 50 – 300 nm), and the monodispersed PS were suitable for subsequent particle number analyses (in LIBD).

7.2. Mass concentration determination

A total organic carbon (TOC) analyzer was used to determine the mass concentration of PS standards as non-purgeable organic carbon content, but 100% conversion efficiency (ratio of measured mass concentration to expected mass concentration) is not guaranteed, especially for large particles [39]. The calibration of TOC analyzer and conversion efficiency of PS standard sizes between 20 and 400 nm is shown in **Figure S18**.

The TOC analyzer shows linear relationship between the expected concentration of the TOC standard (potassium hydrogen phthalate) and for the range of 0.2 – 10 mgC L^{-1}. The 0.2 mgC L^{-1} concentration is the detection limit attained for the analysis protocol in use [40]. The carbon concentration determined with the TOC analyzer for PS 20 – 400 nm is close to the expected carbon concentration, indicating a conversion efficiency of 92 – 118%. The deviation from 100% could be attributed to either incomplete catalytic combustion of PS NPs into CO_2 during the analysis, or the variation in actual concentration of the supplied suspensions. These two factors could not be distinguished.

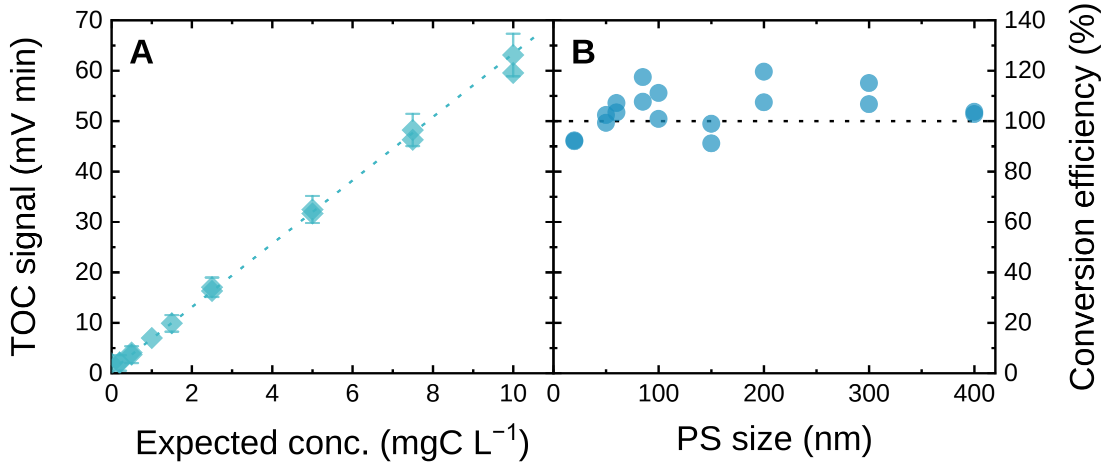

Figure S18. A – Calibration of TOC analyzer with TOC standard (potassium hydrogen phthalate) with a mass concentration range of 0.1 – 10 mgC L^{-1}. B − Conversion efficiency of PS 20 − 400 nm determined with the TOC protocol for organic matter analysis (with an injection volume of 100 µL). Error bars in A are standard deviations from > 40 calibration repeats with the same instrument within the course of 2 years (various projects). Conditions in B: 1 mM $NaHCO_3$, 10 mM NaCl, pH 8.2 ± 0.1.

7.3. Particle number determination

To evaluate the particle counting capability of NTA; the particle count results from NTA were compared with the expected values (from supplier) and the values calculated from mass concentrations (**Figure S19**).

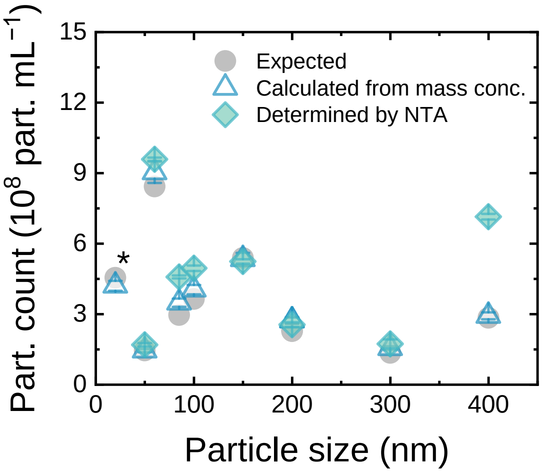

Figure S19. Expected particle numbers (based on information of the supplied standard samples), particles numbers calculated from mass concentrations, and particle numbers determined by NTA. *: NTA data is not included as the PS size (20 nm) is below the detection range.

The particle counts calculated from mass concentrations (TOC results) showed good consistency with expected particle numbers. For 50 − 300 nm particles, NTA results were also consistent with expected and calculated particle counts. In summary, NTA is a reliable method to quantify PS standards.

8. Analytical protocol

The analytical protocol is described in **Table S3**, which includes instrument cleaning before the measurement, instrument configuration, sample analysis, and instrument cleaning after the analysis.

Data validation was done with 100 nm PS standards at a fixed concentration of 100 μg L^{-1}. The BDP of this standard suspension should be around 0.07 − 0.09.

Table S3. Analytical protocol.

No.	Step	Description	Justification
1	Instrument cleaning pre-analysis	***(If the LIBD instrument has not been switched on)***<br>Switch on the LIBD instrument for and wait at least 2 h for the laser to warm up.<br>Set pump flow rate at 5 mL min^{-1} and flush the cuvette with 0.1 mM HCl, then 0.1 mM NaOH, then warm tap water, and finally Milli-Q water, each step for 10 min.	To wash any residual organic matter off the tubing and cuvette. The dead volume from the inlet of capillary to the cuvette outlet is ~ 2 mL.
2	Instrument configuration	Submerge the inlet of capillary in the sample solution (at least 100 mL), cover the flask with aluminum foil.<br>Set the pump flow rate at 1 mL min^{-1}. Set the default number of pulses to 3000 in LIBD LabView software.<br>***For measurements with varied laser pulse energies:*** set the initial laser pulse energy to minimal (1 − 3 μJ); and step size to 3 − 5 μJ.<br>***For measurements with a fixed laser pulse energy:*** set the initial laser pulse energy to the desired value (240 μJ); and step size to 0 μJ.	The step size of 5 μJ allows ~70 BDP data points to be taken per analysis, which may ensure that the analysis is accurate. The lower step size, the more data points obtained.
3	Sample analysis	Once around 10 mL of sample has passed through the cuvette (*i.e.* 15 min later at a flow rate of 1 mL min^{-1}), start the LIBD measurement.	10 mL volume ensures that the sample in the cuvette is not diluted by residual water.
4	Instrument cleaning post-analysis	Remove the inlet of capillary from the sample solution and submerge it in Milli-Q water.<br>Increase the pump flow rate to 5 mL min^{-1} for 10 min to rinse the tubing and cuvette.	

9. Error analysis

9.1. All error sources involved in LIBD calibration

All the error sources involved in the are summarized in **Table S4**. The main source of error comes from the intrinsic analytical error of LIBD.

Table S4. Sources of errors and justification of error weights.

**Parameter**	**Error source**	**Error determination and justification**
Nanoparticle/ Nanoplastic concentration (mg L^{-1} or particles mL^{-1})	Tolerance of analytical balance<br><br>Pipetting (with 5, 1, and 0.2 mL pipettes)<br><br>Volumetric flask<br><br>Human error / sample contamination<br><br>TOC analysis	Balance has very low tolerance (0.1 mg or < 0.1 %).<br><br>Pipette tolerance ~ 1 % (water volume was checked on each day with water mass on a microbalance)<br><br>Volumetric flask tolerance 100.00 ± 0.05 mL<br>→ Preparation results in < 1 % uncertainty in calibration solution concentration.<br><br>Solution (including background electrolytes) was prepared the same day as analysis.<br><br>TOC analysis for determining the PS concentration causes 5 % error.
Solution temperature	Ambient temperature	± 2 °C. Measured several times per day.<br><br>Thermocouple is accurate to 0.1 °C and induce low intrinsic temperature error.
Flow rate	Temperature / Water density<br><br>Pump pulsation (not measured)	Has negligible contribution on LIBD analytical error, see **Figure S6**.
Feed solution pH	Weighing of NaCl and $NaHCO_3$ powders<br><br>Volumetric flask (1 L)<br><br>Human error	8.2 ± 0.1, controlled *via* the use of buffers 1 mM $NaHCO_3$ and 10 mM NaCl
LIBD analysis	Variation of laser pulse energy<br><br>Variation of acoustic signals at a given concentration<br><br>Conversion from BDP to particle concentration	The variation of laser pulse energy is controlled at ± 3 µJ.<br><br>40 measurements allow determination of standard deviation of each solution. Relative error (standard deviation / value) may amount 10 – 50 % depending on BDP and the shape of S-curve.<br><br>BDP – concentration conversion was done for all 40 measurements per sample, then standard deviation was applied.

10. References

[1] US Environmental Protection Agency, Definition and procedure for the determination of the method detection limit, revision 2. https://www.epa.gov/sites/default/files/2016-12/documents/mdl-procedure_rev2_12-13-2016.pdf (accessed 10 November 2021).

[2] Pecora, R., 2000. Dynamic light scattering measurement of nanometer particles in liquids. J. Nanopart. Res. 2(2), 123-131.

[3] Kaszuba, M., McKnight, D., Connah, M.T., McNeil-Watson, F.K., Nobbmann, U., 2008. Measuring sub nanometre sizes using dynamic light scattering. J. Nanopart. Res. 10(5), 823-829.

[4] Rieker, T., Hanprasopwattana, A., Datye, A., Hubbard, P., 1999. Particle size distribution inferred from small-angle x-ray scattering and transmission electron microscopy. Langmuir 15(2), 638-641.

[5] Li, T., Senesi, A.J., Lee, B., 2016. Small angle X-ray scattering for nanoparticle research. Chem. Rev. 116(18), 11128-11180.

[6] Feng, J., Kriechbaum, M., Liu, L., 2019. In situ capabilities of small angle X-ray scattering. Nanotechnology Reviews 8(1), 352-369.

[7] Filipe, V., Hawe, A., Jiskoot, W., 2010. Critical evaluation of nanoparticle tracking analysis (NTA) by NanoSight for the measurement of nanoparticles and protein aggregates. Pharm. Res. 27(5), 796-810.

[8] Gross, J., Sayle, S., Karow, A.R., Bakowsky, U., Garidel, P., 2016. Nanoparticle tracking analysis of particle size and concentration detection in suspensions of polymer and protein samples: Influence of experimental and data evaluation parameters. Eur. J. Pharm. Biopharm. 104, 30-41.

[9] Austin, J., Minelli, C., Hamilton, D., Wywijas, M., Jones, H.J., 2020. Nanoparticle number concentration measurements by multi-angle dynamic light scattering. J. Nanopart. Res. 22(5), 108.

[10] Rossé, P., Loizeau, J.L., 2003. Use of single particle counters for the determination of the number and size distribution of colloids in natural surface waters. Colloids Surf. Physicochem. Eng. Aspects 217(1), 109-120.

[11] Witt, W., Rüthele, S., 1996. Laser diffraction – Unlimited? Particle & Particle Systems Characterization 13(5), 280-286.

[12] Totoki, S., Yamamoto, G., Tsumoto, K., Uchiyama, S., Fukui, K., 2015. Quantitative laser diffraction method for the assessment of protein subvisible particles. J. Pharm. Sci. 104(2), 618-626.

[13] Lee, H., Chen, S.-C., Kim, C., Westenburg, E., Moon, S.I., Pui, D.Y.H., 2017. Evaluation of concentration measurement techniques of colloidal nanoparticles for microfiltration and ultrafiltration applications: Inductively coupled plasma-mass spectrometry, nanoparticle tracking analysis and electrospray-scanning mobility particle sizer. Sep. Purif. Technol. 184, 34-42.

[14] Laborda, F., Bolea, E., Jiménez-Lamana, J., 2014. Single particle inductively coupled plasma mass spectrometry: A powerful tool for nanoanalysis. Anal. Chem. 86(5), 2270-2278.

[15] Montaño, M.D., Badiei, H.R., Bazargan, S., Ranville, J.F., 2014. Improvements in the detection and characterization of engineered nanoparticles using spICP-MS with microsecond dwell times. Environmental Science: Nano 1(4), 338-346.

[16] Lee, S., Bi, X., Reed, R.B., Ranville, J.F., Herckes, P., Westerhoff, P., 2014. Nanoparticle size detection limits by single particle ICP-MS for 40 elements. Environ. Sci. Technol. 48(17), 10291-10300.

[17] Pergantis, S.A., Jones-Lepp, T.L., Heithmar, E.M., 2012. Hydrodynamic chromatography online with single particle-inductively coupled plasma mass spectrometry for ultratrace detection of metal-containing nanoparticles. Anal. Chem. 84(15), 6454-6462.

[18] Xu, Y., Ou, Q., Jiao, M., Liu, G., van der Hoek, J.P., 2022. Identification and quantification of nanoplastics in surface water and groundwater by pyrolysis gas chromatography–mass spectrometry. Environ. Sci. Technol. 56(8), 4988-4997.

[19] Okoffo, E.D., Thomas, K.V., 2024. Quantitative analysis of nanoplastics in environmental and potable waters by pyrolysis-gas chromatography–mass spectrometry. J. Hazard. Mater. 464, 133013.

[20] Lin, Y., Huang, X., Liu, Q., Lin, Z., Jiang, G., 2020. Thermal fragmentation enhanced identification and quantification of polystyrene micro/nanoplastics in complex media. Talanta 208, 120478.

[21] Vladár, A.E., Hodoroaba, V.-D., 2020. Characterization of nanoparticles by scanning electron microscopy, Characterization of Nanoparticles. Elsevier, Walthm, MA, USA, pp. 7-27.

[22] Verleysen, E., Wagner, T., Lipinski, H.-G., Kägi, R., Koeber, R., Boix-Sanfeliu, A., De Temmerman, P.-J., Mast, J., 2019. Evaluation of a TEM based approach for size measurement of particulate (nano)materials. Materials 12(14).

[23] Klapetek, P., Valtr, M., Nečas, D., Salyk, O., Dzik, P., 2011. Atomic force microscopy analysis of nanoparticles in non-ideal conditions. Nanoscale Res. Lett. 6(1), 514-519.

[24] Dazzi, A., Prater, C.B., 2017. AFM-IR: Technology and applications in nanoscale infrared spectroscopy and chemical imaging. Chem. Rev. 117(7), 5146-5173.

[25] DeBlois, R.W., Bean, C.P., 1970. Counting and sizing of submicron particles by the resistive pulse technique. Rev. Sci. Instrum. 41(7), 909-916.

[26] Song, Y., Zhang, J., Li, D., 2017. Microfluidic and nanofluidic resistive pulse sensing: A review. Micromachines 8(7), 204.

[27] Kozak, D., Anderson, W., Vogel, R., Trau, M., 2011. Advances in resistive pulse sensors: Devices bridging the void between molecular and microscopic detection. Nano Today 6(5), 531-545.

[28] Willmott, G.R., Vogel, R., Yu, S.S.C., Groenewegen, L.G., Roberts, G.S., Kozak, D., Anderson, W., Trau, M., 2010. Use of tunable nanopore blockade rates to investigate colloidal dispersions. J. Phys.: Condens. Matter 22(45), 454116.

[29] Roberts, G.S., Yu, S., Zeng, Q., Chan, L.C.L., Anderson, W., Colby, A.H., Grinstaff, M.W., Reid, S., Vogel, R., 2012. Tunable pores for measuring concentrations of synthetic and biological nanoparticle dispersions. Biosensors and Bioelectronics 31(1), 17-25.

[30] Burg, T.P., Godin, M., Knudsen, S.M., Shen, W., Carlson, G., Foster, J.S., Babcock, K., Manalis, S.R., 2007. Weighing of biomolecules, single cells and single nanoparticles in fluid. Nature 446(7139), 1066-1069.

[31] Panchal, J., Kotarek, J., Marszal, E., Topp, E.M., 2014. Analyzing subvisible particles in protein drug products: A comparison of dynamic light scattering (DLS) and resonant mass measurement (RMM). AAPS Journal 16(3), 440-451.

[32] Scherbaum, F.J., Knopp, R., Kim, J.I., 1996. Counting of particles in aqueous solutions by laser-induced photoacoustic breakdown detection. Appl. Phys. B 63(3), 299-306.

[33] Walther, C., 2003. Comparison of colloid investigations by single particle analytical techniques—a case study on thorium-oxyhydroxides. Colloids Surf. Physicochem. Eng. Aspects 217(1), 81-92.

[34] Tröster, M., Lipp, P., Sacher, F., Hofmann, T., Brauch, H.J., 2014. Application of laser-induced breakdown-detection as a sensitive detector for UF membrane surrogate challenge tests. Water Supply 15(2), 377-383.

[35] ISO, ISO 22412:2008: Particle size analysis — Dynamic light scattering (DLS). https://www.iso.org/standard/65410.html (accessed 19 February 2023).

[36] Bundschuh, T., Knopp, R., Kim, J.I., 2001. Laser-induced breakdown detection (LIBD) of aquatic colloids with different laser systems. Colloids Surf. Physicochem. Eng. Aspects 177(1), 47-55.

[37] Walther, C., Bitea, C., Hauser, W., Kim, J.I., Scherbaum, F.J., 2002. Laser induced breakdown detection for the assessment of colloid mediated radionuclide migration. Nuclear Instruments and Methods in Physics Research Section B: Beam Interactions with Materials and Atoms 195(3), 374-388.

[38] Walther, C., Cho, H.R., Fanghänel, T., 2004. Measuring multimodal size distributions of aquatic colloids at trace concentrations. Appl. Phys. Lett. 85(26), 6329-6331.

[39] Schmidtmann, J., Peiffer, S., 2024. A rapid method to quantify sub-micrometer polystyrene particles in aqueous model systems by TOC analysis. Microplastics and Nanoplastics 4(1), 1-6.

[40] Imbrogno, A., Nguyen, M.N., Schäfer, A.I., 2024. Tutorial review of error evaluation in experimental water research at the example of membrane filtration. Chemosphere 357, 141833.
